# Interleukin‐17 regulates matrix metalloproteinase activity in human pulmonary tuberculosis

**DOI:** 10.1002/path.5013

**Published:** 2018-01-18

**Authors:** Shivani Singh, George Maniakis‐Grivas, Utpal K Singh, Radha M Asher, Francesco Mauri, Paul T Elkington, Jon S Friedland

**Affiliations:** ^1^ Infectious Diseases and Immunity Imperial College London UK; ^2^ Tuberculosis Unit, Department of Medicine Nalanda University Hospitals Agam Kuan Patna India; ^3^ Department of Histopathology, Hammersmith Hospitals Imperial College London UK

**Keywords:** mycobacteria, MMP, innate immunity, T_H_‐17, immunopathology

## Abstract

Tuberculosis (TB) is characterized by extensive pulmonary matrix breakdown. Interleukin‐17 (IL‐17) is key in host defence in TB but its role in TB‐driven tissue damage is unknown. We investigated the hypothesis that respiratory stromal cell matrix metalloproteinase (MMP) production in TB is regulated by T‐helper 17 (T_H_‐17) cytokines. Biopsies of patients with pulmonary TB were analysed by immunohistochemistry (IHC), and patient bronchoalveolar lavage fluid (BALF) MMP and cytokine concentrations were measured by Luminex assays. Primary human airway epithelial cells were stimulated with conditioned medium from human monocytes infected with Mycobacterium tuberculosis (Mtb) and T_H_‐17 cytokines. MMP secretion, activity, and gene expression were determined by ELISA, Luminex assay, zymography, RT‐qPCR, and dual luciferase reporter assays. Signalling pathways were examined using phospho‐western analysis and siRNA. IL‐17 is expressed in TB patient granulomas and MMP‐3 is expressed in adjacent pulmonary epithelial cells. IL‐17 had a divergent, concentration‐dependent effect on MMP secretion, increasing epithelial secretion of MMP‐3 (p < 0.001) over 72 h, whilst decreasing that of MMP‐9 (p < 0.0001); mRNA levels were similarly affected. Both IL‐17 and IL‐22 increased fibroblast Mtb‐dependent MMP‐3 secretion but IL‐22 did not modulate epithelial MMP‐3 expression. Both IL‐17 and IL‐22, but not IL‐23, were significantly up‐regulated in BALF from TB patients. IL‐17‐driven MMP‐3 was dependent on p38 MAP kinase and the PI3K p110α subunit. In summary, IL‐17 drives airway stromal cell‐derived MMP‐3, a mediator of tissue destruction in TB, alone and with monocyte‐dependent networks in TB. This is regulated by p38 MAP kinase and PI3K pathways. © 2017 The Authors. *The Journal of Pathology* published by John Wiley & Sons Ltd on behalf of Pathological Society of Great Britain and Ireland.

## Introduction

*Mycobacterium tuberculosis* (Mtb) has evolved adaptive mechanisms to evade host immunity with such success that today it is the number 1 infectious killer in the world and caused 1.3 million deaths in 2016 (http://www.who.int/tb/publications/global_report/en/). Cavities are pivotal to the spread of TB but the mechanisms that drive them are less well understood 1. Diverse studies indicate that host matrix metalloproteinases (MMPs) are key 2, 3 and we recently showed that collagen destruction may be a key initial event triggering caseous necrosis 4. MMPs can degrade all ECM components including type I collagen at neutral pH 5. High MMP‐9 concentrations correlated with disease severity and the presence of granulomas in tuberculous pleurisy 6. A matrix‐degrading phenotype in human TB where MMP activity was relatively unopposed by specific tissue inhibitors of matrix metalloproteinases (TIMPs) was first described by our group 7. MMP‐1 and MMP‐3 concentrations are increased in respiratory secretions of TB patients 8. In the rabbit model, MMP‐1 expression was greater in TB cavities and an MMP‐1/TIMP imbalance was associated with the development of cavities containing very high bacterial burdens 9. A study in the zebrafish model showed that MMP‐9 from epithelial cells, induced by the mycobacterial virulence factor ESAT‐6, enhanced macrophage recruitment 10, thereby implicating MMPs in immunoregulatory as well as tissue destructive roles in TB.

Respiratory epithelial and other stromal cells are central in host defence to Mtb in addition to phagocytic cells such as alveolar macrophages. Respiratory epithelial cells secrete inflammatory mediators such as chemokines 11, 12, interferon‐γ (IFN‐γ), and anti‐microbial human β‐defensins 13. MMP‐1 and MMP‐9 secretion from bronchial epithelial cells is up‐regulated via TB‐dependent cellular networks 14. Another stromal cell, the lung fibroblast, also secretes mediators that limit the local growth of Mtb 15. Such networks between leukocytes and stromal cells appear key, both in host defence and in driving innate inflammatory tissue damage.

T‐helper 1 (T_H_‐1) cells have historically been thought to be necessary in the control of Mtb infection but T_H_‐17 cells have now been identified as pivotal in Mtb control 16. IL‐17 is a pro‐inflammatory cytokine that functions via mesenchymal and myeloid cells to induce the secretion of diverse cytokines, chemokines, anti‐microbial peptides, and MMPs 17, 18, 19. Mice with a genetically inactivated IL‐17 receptor were unable to exert long‐term control of Mtb, despite a functional T_H_‐1 response 20. IL‐17 knockout mice failed to develop mature granulomas in bacillus Calmette–Guérin (BCG)‐infected lung and had impaired protection from virulent Mtb 21. Although IL‐17 is dispensable for immunity against laboratory‐adapted strains of Mtb, infection with the hypervirulent W‐Beijing strain HN878 required IL‐17 for early immunity 22. In cynomolgus macaques, sterile granulomas had a higher frequency of T cells producing IL‐17 23 and pulmonary delivery of BCG vaccine triggered a mucosal immune response orchestrated by IL‐17 24. Finally, in a study in the Chinese Han population, genetic polymorphisms in IL‐17A and IL‐17F were associated with host susceptibility to TB 25.

IL‐22, a second T_H_‐17 family cytokine, has both anti‐ and pro‐inflammatory activity at mucosal interfaces 26. IL‐22 produced by human NK cells inhibits the growth of Mtb by enhancing phagolysosomal fusion due to enhanced expression of calgranulin A 27. IL‐22 was also produced by human NK cells in TB pleural fluid in response to BCG and Mtb‐related antigens, suggesting that it might participate in the recall immune response for Mtb infection 28. IL‐17 and IL‐22 function synergistically to induce epithelial mediators such as human β‐defensin‐2, S100 and lipocalin‐2 29. IL‐23, a member of the IL‐12 family, is essential for stabilization and polarization of lymphocytes towards a T_H_‐17 phenotype and promotes IL‐17 secretion by activated T_H_‐17 cells 30. *IL23A* mRNA was up‐regulated in unfractionated BAL cells from TB patients compared with controls 31 and pulmonary IL‐23 gene delivery with a vaccine adjuvant augmented the expansion of Mtb‐specific CD4^+^ T cells which produced IL‐17 32, 33, with a simultaneous reduction in mycobacterial burden and pulmonary inflammation.

Therefore, accumulating evidence suggests a central role for T_H_‐17 cytokines in host–pathogen interactions in TB. We hypothesized that in TB‐dependent networks, stromal cell MMP secretion is regulated by T_H_‐17 cytokines. First, we identified IL‐17 expression in lymphocytes around pulmonary granulomas in TB patients. Next, we showed that IL‐17 increased mRNA expression and secretion of epithelial MMP‐3, which was highly expressed in respiratory epithelial cells in TB patients. In contrast, IL‐17 decreased respiratory epithelial cell MMP‐9 production. Both IL‐17 and IL‐22 increased fibroblast MMP‐3 secretion and we demonstrated for the first time that both of these cytokines are elevated in BALF from TB patients. Finally, we investigated the mechanisms involved in IL‐17 signalling and show that p38 mitogen‐activated protein kinase (MAPK) and the p110α subunit phosphoinositide 3‐kinase (PI3K) signalling paths are key.

## Materials and methods

Further details, including experimental design and statistical methods, may be found in the supplementary material, Supplementary materials and methods.

### Immunohistochemical analysis of patient biopsies

The project was approved by the Hammersmith and Queen Charlotte's and Chelsea Research Ethics Committee, London (ref 07/H0707/120). Immunohistochemistry (IHC) was performed on paraffin‐embedded lung biopsies. Antibodies were purchased from Abcam, Cambridge, UK.

### Clinical study

BALF samples were collected from patients being routinely investigated for respiratory symptoms at Nalanda University Hospitals, Patna, India. The study was approved by the ethics review board at Nalanda Medical College and University Hospitals (ref SS/0810/TB). Samples were centrifuged and sterile‐filtered to remove cellular debris and Mtb 34.

### Monocyte purification, infection, and generation of CoMTb

Primary blood mononuclear cells (PBMCs) were from two donor buffy coats from healthy donors (National Blood Transfusion Service, UK). Monocytes were infected with the Mtb H37Rv strain at a multiplicity of infection (MOI) of 1. For epithelial cells, Ziehl–Nielsen staining demonstrated that 30% cells were infected at a MOI of 10, which is similar to previous reports 35. Medium from infected monocytes was termed conditioned medium from monocytes infected with Mtb (CoMTb). Control medium was generated in an identical manner but without infection and was called CoMCont.

### Cell culture and experimental design

Primary small airway epithelial cells (SAECs) and normal human bronchial epithelial (NHBE) cells were cultured in bronchial epithelial growth media (Lonza Biosciences, Basel, Switzerland) and human MRC‐5 fibroblasts were grown in Eagle's medium (Sigma‐Aldrich, Gillingham, Dorset, UK), according to the suppliers' instructions. Epithelial cells were stimulated with a 1 in 5 dilution of CoMTb and MRC‐5 cells with a 1 in 50 dilution. Supernatants were harvested at 72 h for secretion analysis and mRNA extraction was performed at 24 h 14, 36.

### Promoter‐reporter assay

Promoter‐reporter studies were performed using FuGENE HD Transfection Reagent and Promega's Dual‐Luciferase Reporter Assay System (Promega‐UK, Southampton, UK). The MMP‐3 promoter (1206 base pairs) was linked to firefly luciferase and the reference gene thymidine kinase promoter was linked to *Renilla* luciferase.

### Phospho‐western analysis and gelatin zymography

After electrophoresis, proteins were electro‐transferred to nitrocellulose membranes and probed with a primary antibody, then washed and incubated with a secondary antibody. Luminescence was produced using the ECL system. MMP‐9 gelatinolytic activity was detected by zymography using standard methodology 37.

### Small interfering RNA (siRNA) transfection

All siRNAs pools targeted the transcription products from four alleles of the gene of interest. Conditions for transfection were optimized using a transfection control and a negative control (non‐targeting siRNA; supplementary material, Figure S1).

### RNA extraction, cDNA synthesis, and reverse transcription–quantitative polymerase chain reaction (RT‐qPCR)

RNA extraction was performed using the Qiagen RNeasy Minikit (QIAGEN Ltd, Manchester, UK). cDNA synthesis was performed using a Qiagen Quantitect reverse transcription kit (QIAGEN Ltd). Real‐time quantitative PCR was performed using the Brilliant II QPCR master mix on the Stratagene Mx3000P platform (Stratagene, Cambridge, UK). MMP primers and probes have been described previously 38. Experimental MMP data were normalized to three reference genes. Analysis of *MMP* mRNA expression was first undertaken by the standard curve method, and results were corroborated by C_T_ values assessing levels of gene expression.

## Results

### IL‐17 is expressed in human TB granulomas and has divergent effects on epithelial cell MMP‐3 and MMP‐9 secretion

**Figure 1:**
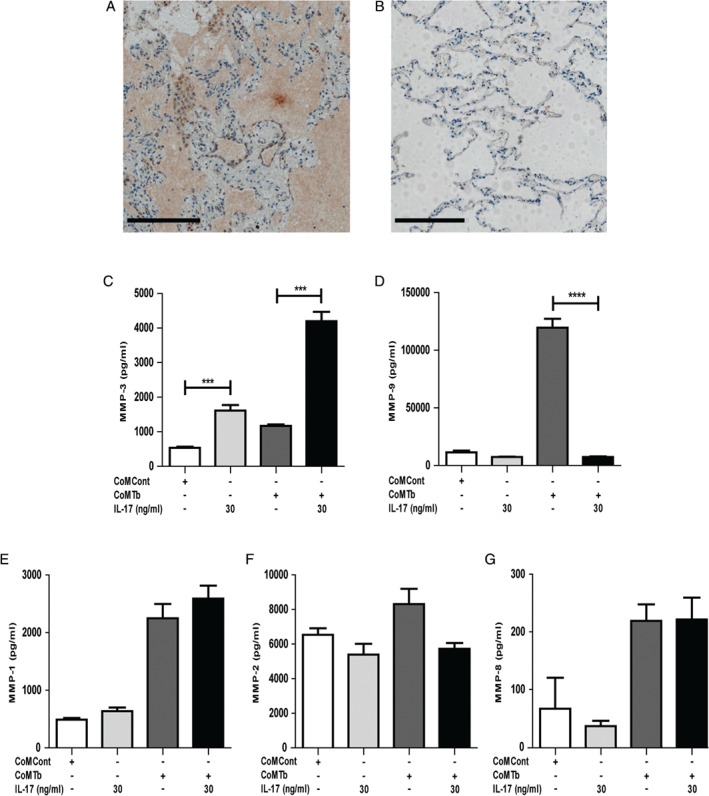
IL‐17 is expressed in granulomas of TB patients and has a divergent effect on epithelial MMP secretion in a TB network. (A) IL‐17 is expressed around granulomas in patients with TB. The figure is representative of lung biopsies from five patients with Mtb infection (scale bar = 200 μm). (B) Control normal lung tissue showed no immunoreactivity for IL‐17 (scale bar = 200 μm). (C) MMP‐3 secretion increased 2.2‐fold after stimulation with CoMTb and this was further augmented four‐fold by IL‐17. IL‐17 also increased the baseline secretion of MMP‐3 from SAECs by three‐fold (p < 0.001). (D) CoMTb increased MMP‐9 secretion 10.5‐fold (p = 0.002), but this was suppressed by IL‐17 (p < 0.0001). (E) MMP‐1 secretion from SAECs was augmented 4.5‐fold by CoMTb (p < 0.001), but was not altered by IL‐17. (F) SAECs constitutively secrete MMP‐2, which was not altered significantly by CoMTb. (G) MMP‐8 was augmented by CoMTb but only at very low concentrations. In the figures, * indicates p < 0.05, **p < 0.01, ***p < 0.001, and ****p < 0.0001.

First, we investigated the expression of IL‐17 in five TB and five control human lung biopsy specimens. IL‐17 was expressed in TB granulomas (Figure 1A) but not in control lung biopsies of normal tissue (Figure 1B). As a positive control, we detected colonic T‐lymphocyte immunoreactivity (supplementary material, Figure S2A). No staining was seen when the primary antibody was omitted as a negative control (supplementary material, Figure S2B). Next, we investigated MMP secretion from human distal small airway epithelial cells (SAECs). MMP‐1, MMP‐3, and MMP‐9 secretion from SAECs was all increased following stimulation by CoMTb at a 1 in 5 dilution. MMP‐3 secretion increased 2.2‐fold from 534 ± 34 pg/ml to 1171 ± 44 pg/ml (Figure 1C, *p* = 0.005) and MMP‐9 secretion increased 10.5‐fold from 11 446 ± 15 pg/ml to 119 561 ± 77 pg/ml (Figure 1D, *p* = 0.002). MMP‐1 secretion increased 4.5‐fold from a baseline of 490 ± 30 pg/ml to 2247 ± 25 pg/ml (Figure 1E, *p* = 0.008). SAECs constitutively secrete MMP‐2 at a baseline which was not altered significantly by CoMTb (Figure 1F). MMP‐8 was detectable but only at very low concentrations (Figure 1G). MMP‐7, MMP‐12, and MMP‐13 were almost undetectable (data not shown).

Next, we investigated the effect of IL‐17 on MMP secretion. In SAECs, maximal MMP‐3 secretory effect was observed with 30 ng/ml IL‐17 (supplementary material, Figure S3A) 39, 40. In co‐stimulation experiments with CoMTb, IL‐17 increased baseline epithelial MMP‐3 secretion from 534 ± 34 pg/ml to 1612 ± 17 pg/ml (*p* < 0.001), and CoMTb‐driven MMP‐3 secretion from 1171 ± 44 pg/ml to 4196 ± 28 pg/ml (Figure 1C, *p* < 0.001). In contrast, CoMTb‐driven MMP‐9 secretion was suppressed by IL‐17 from 119 561 ± 77 pg/ml to 7483 ± 50 pg/ml (Figure 1D, *p* < 0.0001). MMP‐9‐induced gelatinolysis, measured by zymography, was similarly decreased by IL‐17 (data not shown). Changes in gene expression were consistent with secretion data (data not shown). IL‐17 did not alter MMP‐1, MMP‐2 or MMP‐8 secretion from SAECs (Figure 1E–G). Lastly, we investigated TIMP‐1/2 secretion as the MMP/TIMP ratio is functionally important in determining net matrix degradation. CoMTb suppressed TIMP‐1 secretion (supplementary material, Figure S3B, *p* < 0.5) but IL‐17 did not significantly modulate this. TIMP‐2 secretion was also unaffected by IL‐17 (supplementary material, Figure S3C); therefore, these results confirm that up‐regulation of MMP‐3 in TB is unopposed by inhibitor secretion.

### MMP‐3 is expressed by epithelial cells in TB and secretion is up‐regulated by IL‐17 in a TB network, whereas direct infection with Mtb does not modulate expression

**Figure 2:**
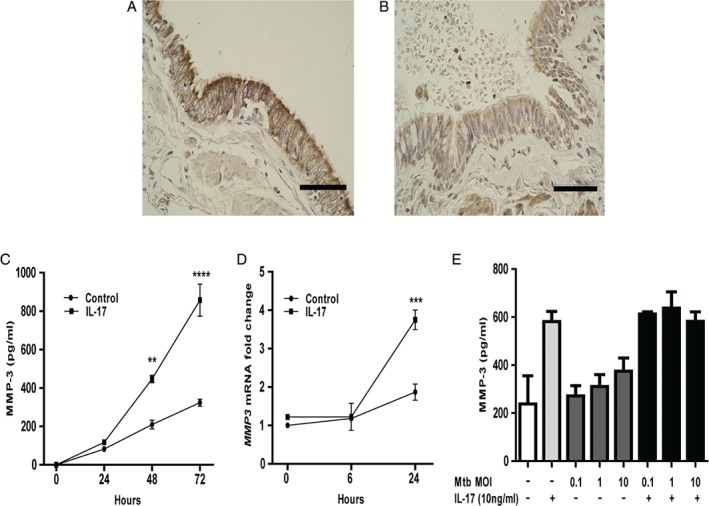
MMP‐3 is expressed in human respiratory epithelial cells in TB patients and MMP‐3 gene expression and secretion are driven by IL‐17. (A) Pulmonary epithelial cells adjacent to TB granulomas express MMP‐3. The figure is representative of lung biopsies from five patients with TB (scale bar = 20 μm). (B) Minimal staining for MMP‐3 was observed in five normal lung specimens (scale bar = 20 μm). (C) Kinetic experiments in NHBE cells demonstrated that MMP‐3 secretion increased progressively until 72 h after stimulation with 10 ng/ml IL‐17 (p < 0.001). (D) IL‐17‐driven MMP‐3 mRNA accumulation peaked at 24 h, rising to four‐fold above baseline (p < 0.01). (E) Direct infection of NHBE cells with Mtb at an MOI from 0.1 to 10 did not alter MMP‐3 secretion and there was no synergy between Mtb infection and IL‐17 stimulation.

In TB patient lung biopsies, pulmonary epithelial cells adjacent to TB granulomas strongly expressed MMP‐3 (Figure 2A), whereas minimal staining was observed in normal lung biopsies (Figure 2B). After confirming that upper airway epithelial cells (NHBE) and SAECs had similar secretion profiles, we performed all further experiments on NHBE cells. Our immunohistochemical studies showed MMP‐3 expression by epithelial cells in the relatively larger airways, which we felt were best modelled by NHBE cells, and hence we used these for further studies. In NHBE cells, maximal MMP‐3 secretory effect was observed with 10 ng/ml IL‐17 (supplementary material, Figure S3D) 41, 42. Kinetic experiments in NHBE cells showed that the MMP‐3 concentration peaked at 72 h when stimulated with IL‐17, after which it declined (Figure 2C, *p* < 0.001), and IL‐17‐driven *MMP‐3* mRNA accumulation peaked at 24 h (Figure 2D, *p* < 0.01). These kinetics are similar to our published data on MMP‐1 and MMP‐9 secretion 14, 36. Next, we investigated the regulation of MMP‐3 and MMP‐9 by IL‐22 and IL‐23 and found no change (supplementary material, Figure S4A–D). Direct infection with Mtb at MOI from 0.1 to 10 had no significant effect on MMP‐3 secretion from NHBE cells in the presence or absence of IL‐17 (Figure 2E). Infection at a higher MOI did not increase the uptake of bacteria and MMP production remained unchanged (data not shown).

To further dissect the IL‐17 and CoMTb‐driven MMP‐3 up‐regulation, we performed cytokine and chemokine analyses in the supernatants from NHBE cells stimulated with IL‐17 alone or in combination with CoMTb. The cytokines and chemokines analysed were IL‐1β, IL‐6, IL‐10, TNF‐α, IL‐1Ra, IFN‐α, IFN‐γ, IL‐13, IL‐15, IL‐17, IL‐12, IL‐5, IL‐2, IL‐7, IL‐2R, IL‐4, RANTES, eotaxin, MIP‐1β, MIP‐1α, MCP‐1, IP‐10, MIG, and CXCL‐8. We found that although TNF‐α, IL‐1RA, CXCL‐8, and MIP‐1α were elevated in both groups, only the CXCL‐8 concentration was significantly different between groups (supplementary material, Table S1, *p* < 0.001). Stimulating NHBE cells with TNF‐α alone and in the presence of IL‐17 did not drive MMP‐3 secretion (supplementary material, Figure S5). These experiments demonstrate that the synergy between multiple mediators drives maximal MMP‐3 production from stromal cells in TB.

### IL‐17 and IL‐22 augment TB‐dependent MMP‐3 secretion from fibroblasts

**Figure 3:**
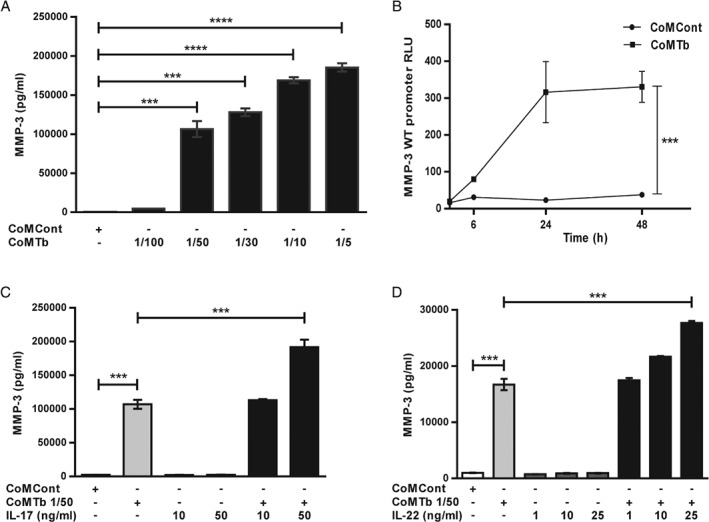
IL‐17 and IL‐22 increase TB‐dependent MMP‐3 secretion from fibroblasts. (A) CoMTb caused a concentration‐dependent increment in MMP‐3 secretion from MRC‐5 fibroblasts (p < 0.01). (B) MMP‐3 full‐length promoter activation in response to CoMTb was detectable at 6 h, peaking at 24 h (p < 0.001), and thereafter remaining stable for 48 h. Results are expressed as relative luminescence (RLU) for firefly/Renilla luciferase. (C) IL‐17 and (D) IL‐22 both increased CoMTb‐driven MMP‐3 secretion from MRC‐5 fibroblasts in a concentration‐dependent manner (p < 0.001 for both cytokines), but neither affected MMP‐3 secretion as a single stimulus.

Next, we investigated MMP secretion from fibroblasts, stromal cells that can secrete high concentrations of MMPs 43. CoMTb stimulation drove a concentration‐dependent increase in MMP‐3 secretion from MRC‐5 fibroblasts at 72 h (Figure 3A). Although maximal MMP‐3 secretion was observed at a CoMTb dilution of 1 in 5 (*p* < 0.0001), this was accompanied with significant cell death. In subsequent experiments, CoMTb was used at a dilution of 1 in 50, which also significantly up‐regulated MMP‐3 secretion (*p* < 0.01) with no effect on cell viability. To investigate further, promoter‐reporter studies were performed using a wild‐type construct of the MMP‐3 promoter. Activation in response to CoMTb was detectable at 6 h, peaking at 24 h, and thereafter remaining stable for 48 h (Figure 3B, *p* < 0.001).

Next, MRC‐5 fibroblasts were stimulated with IL‐17 alone and in combination with CoMTb. IL‐17 further up‐regulated CoMTb‐induced MMP‐3 secretion from 107 005 ± 6729 pg/ml to 191 829 ± 11057 pg/ml (Figure 3C, *p* < 0.001), but did not affect MMP‐3 as a single stimulus. Similarly, IL‐22 almost doubled fibroblast MMP‐3 secretion from 16 694 ± 1734 pg/ml to 27 638 ± 483 pg/ml (Figure 3D, *p* < 0.001), but did not affect baseline MMP‐3 secretion in the absence of CoMTb.

### IL‐17 and IL‐22 are up‐regulated in TB patient bronchoalveolar lavage fluid

**Figure 4:**
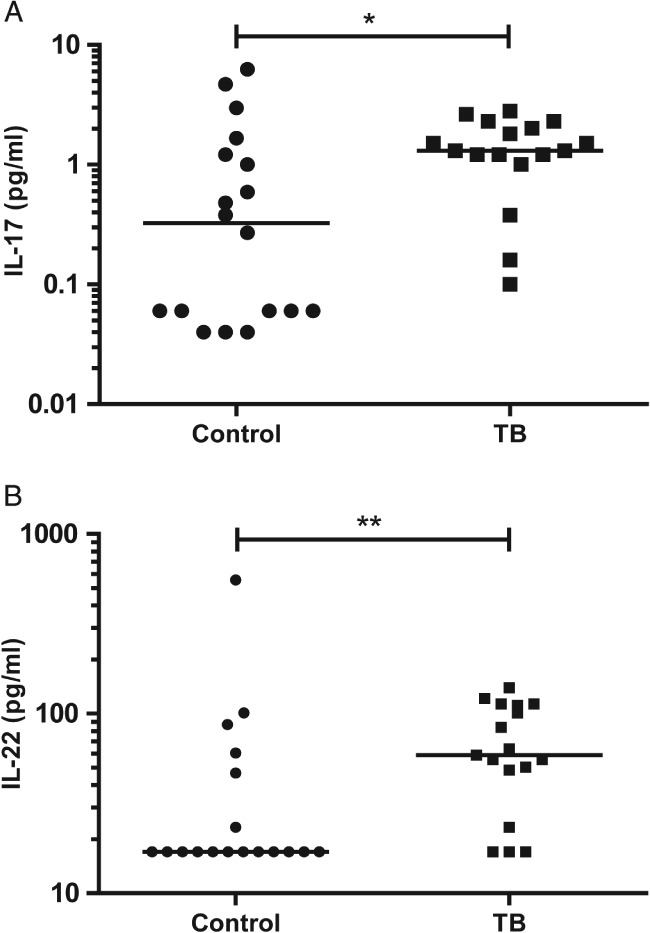
IL‐17 and IL‐22 are up‐regulated in BALF from patients with TB. Concentrations of IL‐17 and IL‐22 were measured in BALF from 17 TB subjects and 18 well‐matched respiratory symptomatic patients. (A) Median IL‐17 concentration was increased in TB patients compared with other respiratory symptomatic patients (p = 0.02), although the absolute detectable concentrations were low. (B) Median IL‐22 concentration in TB patients was four‐fold higher than in control patients (p = 0.006) (Mann–Whitney U‐test).

To examine the relevance of these findings to human TB infection, we investigated IL‐17, IL‐22, and IL‐23 concentrations in BALF from 17 well‐characterized patients with confirmed pulmonary TB and 18 well‐matched respiratory symptomatic patients with other diagnoses that may cause a clinically similar picture, which we have previously reported 44. There were no significant differences between the two groups with regard to age, gender, and smoking history. None of the subjects had a previous history of TB. The median IL‐17 concentration was increased in TB patients compared with other respiratory symptomatic patients (*p* = 0.02), although the absolute concentrations detectable in BALF were low (Figure 4A). The median IL‐22 concentration in BALF from TB patients was four‐fold higher than in control patients (Figure 4B, *p* = 0.006). IL‐23 concentrations were virtually all below the level of detection in both control and TB groups (supplementary material, Figure S6). This demonstrates that the IL‐17 expression seen in lymphocytes by IHC leads to increased concentrations in bronchial lining fluid.

### IL‐17‐driven MMP‐3 secretion is p38 MAPK‐dependent

**Figure 5:**
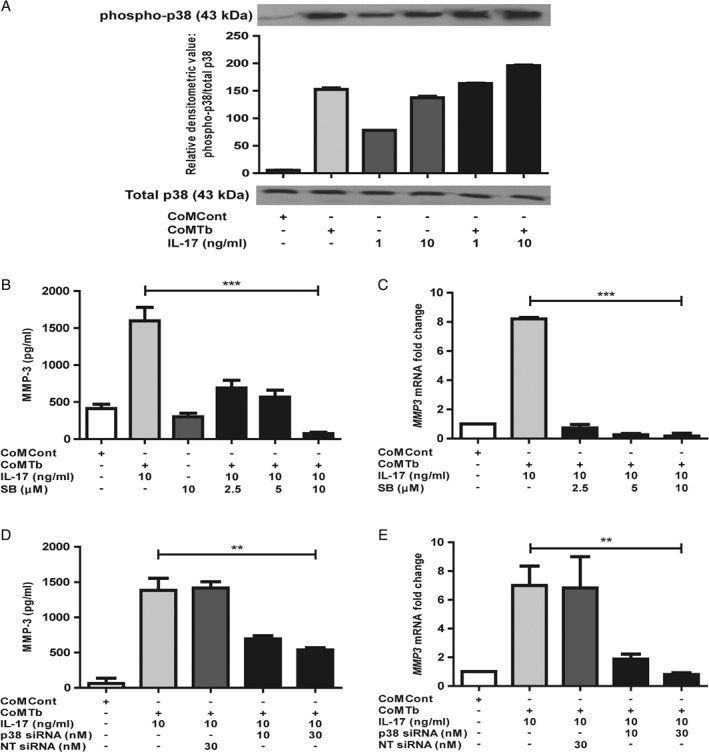
IL‐17 regulation of MMP‐3 secretion is p38 MAP kinase‐dependent. (A) Phosphorylation of p38 after 30 min of stimulation was increased by both CoMTb and IL‐17. p38 phosphorylation was significantly increased when cells were co‐stimulated with IL‐17 and CoMTb. There was no change in total p38. Densitometric analysis was performed using Scion image software. (B) MMP‐3 secretion and (C) MMP‐3 mRNA accumulation driven by IL‐17 was inhibited in a concentration‐dependent manner by SB203580, a specific p38 chemical inhibitor (p < 0.001 for both protein and mRNA). (D) Transfection of the epithelial cells with p38 siRNA decreased IL‐17/CoMTb‐driven MMP‐3 secretion by 50% (p < 0.01). The suppression was maximal with 30 nm of siRNA (E) Similarly, IL‐17‐driven MMP‐3 mRNA decreased to baseline by siRNA inhibition of p38 (p < 0.01). Cells were also transfected with non‐targeting (NT) siRNA, which had no effect.

Next, we investigated the mechanisms regulating IL‐17‐dependent MMP‐3 secretion from NHBE cells. First, we studied the MAPK pathways. Both CoMTb and IL‐17 as single stimuli caused phosphorylation of p38 MAPK, peaking at 30 min, after which it returned to baseline. Phosphorylation of p38 was further increased when cells were co‐stimulated with 10 ng/ml IL‐17 and CoMTb (Figure 5A). Densitometric analysis confirmed increased phosphorylation. ERK 1/2 and JNK MAPKs are constitutively phosphorylated in NHBE cells and were not investigated further.

Next, we investigated the effect of chemical and siRNA‐mediated inhibition of p38 on IL‐17‐dependent MMP‐3 secretion. The p38‐specific chemical inhibitor SB203580 suppressed IL‐17‐driven MMP‐3 secretion in a concentration‐dependent manner to baseline (Figure 5B, *p* < 0.001). Concurrently, inhibition of IL‐17‐dependent *MMP‐3* mRNA accumulation was also observed (Figure 5C, *p* < 0.001). Confirming these data, p38 MAPK‐specific siRNA decreased IL‐17/CoMTb‐driven MMP‐3 secretion by 50% (Figure 5D, *p* < 0.01) and mRNA expression to baseline levels (Figure 5E, *p* < 0.01). The suppression was maximal with 30 nm siRNA. Cells were also transfected with non‐targeting siRNA (NT siRNA) and no change was observed. siRNA knockdown of phospho‐p38 was confirmed by western blotting and at the mRNA level (supplementary material, Figure S7A, B). Significant knockdown was observed at 10 nm p38 siRNA and levels were undetectable with 30 nm siRNA in NHBE cells stimulated with CoMTb and 10 ng/ml IL‐17. Non‐targeting siRNA had no effect on p38 expression. Similar results with p38 chemical and siRNA‐mediated inhibition were observed in SAECs (data not shown). In summary, these data show that p38 MAPK signalling regulates IL‐17‐driven MMP‐3 secretion from NHBE cells in a TB network.

### Epithelial MMP‐3 secretion in TB is regulated by the PI3K p110α subunit and AKT

**Figure 6:**
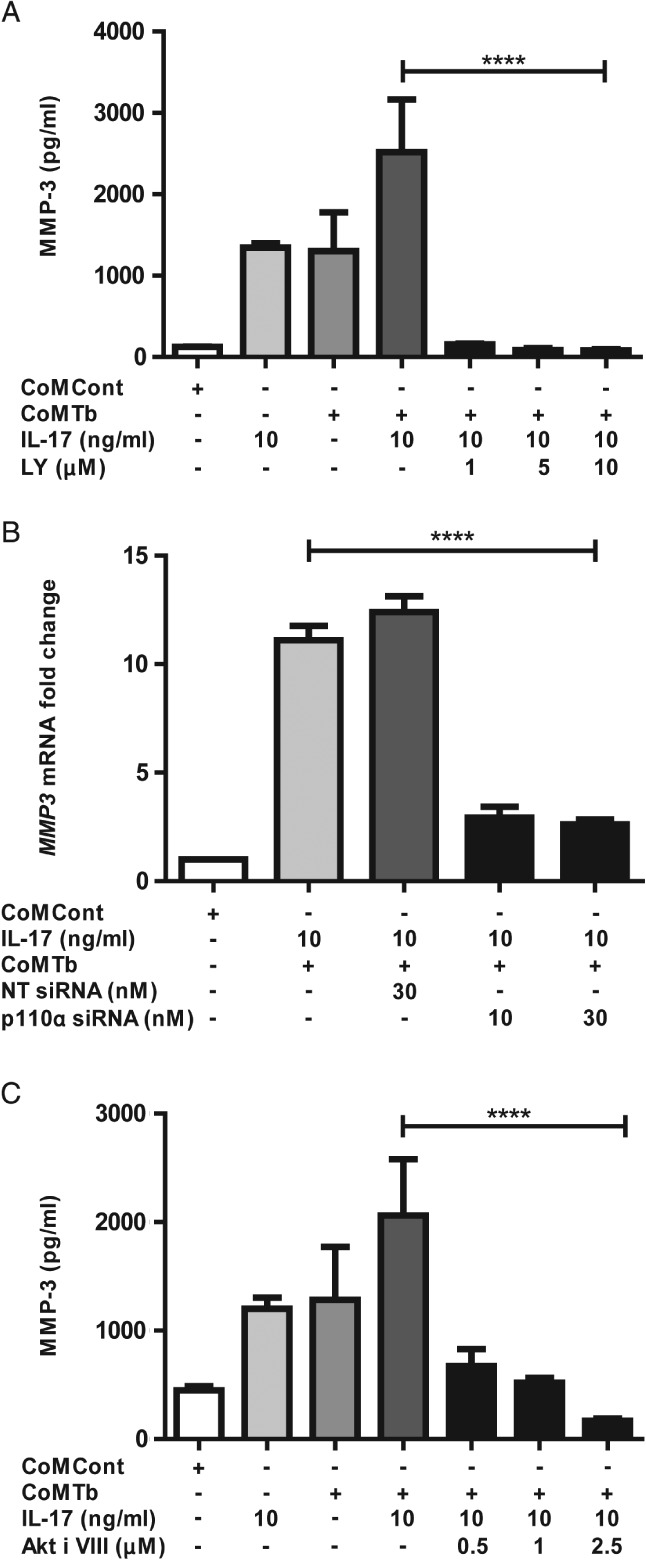
Epithelial MMP‐3 secretion is regulated by the PI3K p110α subunit and AKT. (A) IL‐17‐dependent MMP‐3 secretion was reduced to baseline after incubation of NHBE cells with LY294002, a non‐specific PI3K proximal subunit inhibitor (p < 0.0001). Suppression was maximal at 10 μm. (B) siRNA transfection of NHBE cells identified that the p110α subunit was key in this suppression (p < 0.0001). MMP‐3 mRNA was suppressed to baseline with 30 nm siRNA. Cells were also transfected with non‐targeting (NT) siRNA, which had no effect. (C) Downstream, inhibition of AKT by a specific inhibitor (AKT inhibitor VIII) resulted in MMP‐3 suppression to baseline. The effect was dose‐dependent and maximal at 2.5 μm of the AKT inhibitor (p < 0.0001).

Finally, we investigated whether the PI3‐kinase (PI3K) pathway had a role in IL‐17‐driven MMP‐3 up‐regulation, since this path has been implicated in the IL‐17A‐mediated regulation of several other genes in human airway epithelial cells 39. The non‐specific PI3K proximal catalytic subunit inhibitor LY294002 suppressed IL‐17‐dependent MMP‐3 secretion to control concentrations (Figure 6A, *p* < 0.0001). To identify the specific p110 catalytic subunit involved, NHBE cells were transfected with siRNAs specific for the p110 catalytic subunit isoforms. *MMP‐3* mRNA was suppressed to baseline with the p110α‐specific inhibitor (Figure 6B, *p* < 0.0001). There was no change upon pre‐incubation with non‐targeting siRNA, the negative control. In contrast, p110β subunit‐specific inhibition did not alter MMP production (data not shown). siRNA knockdown of the p110α subunit was confirmed by western blotting and at the mRNA level (supplementary material, Figure S7C, D). Significant knockdown was observed at 10 nm and levels were undetectable with 30 nm siRNA, with no effect seen with non‐targeting siRNA.

As the γ and δ isoforms are almost exclusively expressed in leukocytes, they were not investigated. Downstream of PI3K, inhibition of AKT similarly suppressed MMP‐3 secretion to baseline (Figure 6C, *p* < 0.0001). Thus, both proximal and distal nodes of the PI3K signalling pathway regulate IL‐17‐mediated *MMP‐3* gene expression and secretion in TB.

## Discussion

We have demonstrated for the first time that IL‐17 is expressed within human TB granulomas and that adjacent epithelial cells express MMP‐3. IL‐17 causes up‐regulation of *MMP‐3* gene expression and secretion from epithelial cells exposed to a monocyte‐dependent infection *in vitro* network. Since SAECs and NHBE cells have different immunological roles 45, we first investigated their secretion profiles and identified these to be similar. IHC showed epithelial MMP‐3 expression in the relatively larger airways, which we felt were best modelled by NHBE cells, and hence we used them for further studies. We have previously shown that MRC‐5 fibroblasts and human lung (HL) fibroblasts have similar MMP production profiles in a TB network 43 and therefore we chose to focus on one fibroblast cell line for this study. Both T_H_‐17 cytokines, IL‐17 and IL‐22, drive MMP‐3 secretion from MRC‐5 fibroblasts exposed to CoMTb. In contrast, IL‐17 did not modulate the effects of direct infection by Mtb. The concentrations of both of these cytokines were elevated in BALF from pulmonary TB patients but not from other patients with similar respiratory symptoms. We have defined key mechanisms through which IL‐17 regulates MMP‐3, which is a mediator of tissue damage in TB, in part through its action activating the collagenase MMP‐1 46. Together, these data define a new role for IL‐17 in up‐regulating tissue‐destructive proteases in TB, in addition to previously described functions in granuloma development and control of infection 20, 21.

The action of IL‐17 to increase baseline and CoMTb‐driven epithelial cell *MMP‐3* gene expression and secretion is consistent with other studies. In murine embryonic fibroblasts, IL‐17 regulated chemokine and MMP (MMP‐3, MMP‐9, and MMP‐13) expression to recruit both neutrophils and monocytes 47. IL‐17 and MMP‐3 were expressed in TB lymphocytes and lung epithelial cells, but IL‐17 did not drive epithelial MMP‐3 when cells were directly infected with Mtb. A biologically relevant feature of IL‐17 is its strong cooperative and synergistic effect via mRNA stabilization of other inflammatory cytokines 48, which may account for its effect on MMP‐3 secretion in the TB network between epithelial cells and monocytes. In contrast, CoMTb‐driven MMP‐9 secretion and gene expression were suppressed by IL‐17 and functional activity of MMP‐9 was also reduced. Oriss *et al* 49 recently showed that IL‐23‐dependent IL‐17 gene expression in lung dendritic cells is negatively regulated by MMP‐9 enzymatic activity, suggesting that inhibition of MMP‐9 could facilitate a strong T_H_‐17 response, which is desirable for vaccination against Mtb.

To further dissect the IL‐17 and CoMTb‐driven MMP‐3 up‐regulation, we performed extensive cytokine and chemokine analyses in the supernatants from stimulated NHBE cells and found that only the CXCL‐8 concentration was significantly higher in the TB/IL‐17 network. Stimulating NHBE cells with TNF‐α alone and in the presence of IL‐17 did not drive MMP‐3 secretion. The TNF‐α concentration in CoMTb is 157 pg/ml when used at a 1 in 5 dilution (supplementary material, Table S2). When NHBE cells were stimulated with 5 ng/ml TNF‐α (30‐fold more), this was a weak stimulus to MMP‐9 secretion, alone and in combination with lipoarabinomannan (LAM) 14. Similarly, TNF‐α alone was a weak stimulus to MMP‐1 secretion from epithelial cells 36. In a previous study investigating the direct infection of human macrophages with Mtb, other stimuli such as LPS, BCG, and individual cytokines including TNF‐α, IL‐1β or IFN‐γ did not drive MMP‐1 production 50. Thus, the synergy between different mediators appears key in Mtb‐driven MMP production from stromal cells.

We also demonstrated that MMP‐3 secretion and promoter activity were increased in fibroblasts as a result of a monocyte‐dependent network in TB and that this effect was increased synergistically by both IL‐22 and IL‐17. This is consistent with data in rheumatoid arthritis, where T_H_‐17 cells induced MMP‐1 and MMP‐3 production 51. In human cardiac fibroblasts, IL‐17 stimulated MMP‐1 expression via p38 and ERK‐dependent AP‐1, NF‐κB, and C/EBP‐β activation 52. In the lung, fibroblasts can secrete very high MMP‐1 concentrations 43. IL‐22 did not drive MMP secretion in epithelial cells (supplementary material, Figure S3), which may reflect the divergent signalling paths activated in epithelial cells compared with fibroblasts. Unlike IL‐17, IL‐22 signals mainly via the Stat3 pathway and has a clonogenic and protective effect on human airway epithelial cells 53.

Next, we investigated whether the T_H_‐17 cytokines were elevated in BALF from patients with pulmonary TB. In a study of 17 pulmonary TB patients and 18 matched controls, plasma IL‐17 levels were elevated in patients. IL‐17 levels substantially decreased after treatment and correlated with both CRP and ESR 54. Previous studies showed that IL‐22 may be detected in tuberculous BALF, pleural fluid, and pericardial fluid 55, 56. We confirmed that IL‐22 was significantly up‐regulated in BALF from TB patients and identified for the first time that IL‐17 concentrations were also elevated in TB patients, although concentrations were low (Figure 4). It has been proposed that low levels of soluble IL‐17 in TB are a result of inhibition of T_H_‐17 effectors by the T_H_‐1 effectors at the site of disease 57. However, IL‐17 levels in BALF and pleural fluid, even in the absence of such an inhibitory T_H_‐1 response, are frequently low or undetectable  56, 58, and IL‐17 cell‐mediated inflammation is independent of IL‐22 59. In contrast, *IL‐17* mRNA expression in pleural fluid mononuclear cells was increased by the Mtb peptides ESAT‐6 and culture filtrate protein (CFP)‐10 57. *In situ* hybridization could be used as a further method to confirm increased IL‐17 expression in the lungs of TB patients.

Finally, we dissected key pathways regulating IL‐17‐dependent MMP‐3 secretion. We demonstrated for the first time that inhibition of p38 MAPK, the PI3K p110α subunit, and AKT resulted in the abrogation of IL‐17‐driven MMP‐3 up‐regulation in a TB network. This finding is consistent with studies which showed that IL‐17 regulates mucosal neutrophil immunity via chemokines and growth factors 40. It has been shown in human airway epithelial cells that IL‐17‐mediated induction of IL‐19, CXCL‐1, CXCL‐2, CXCL‐3, CXCL‐5, and CXCL‐6 human defence genes was mediated via the PI3K pathway 39. In airway epithelial cells, regulation of MMPs in Mtb‐dependent respiratory networks was identified to be via the distal rapamycin‐sensitive PI3K/p70^S6K^ cascade, with the proximal p110α subunit augmenting MMP production 60. IL‐17 may enhance the migration of periodontal ligament fibroblasts by increasing MMP‐1 expression through p38 MAPK and NFκB signal transduction pathways 61. IL‐17 has been shown to limit Mtb‐driven HIF‐1α expression 62, showing that there is a complex interplay between inflammatory signalling, hypoxia, and tissue destruction 63.

In summary, we investigated the effect of T_H_‐17 cytokines on epithelial MMP production by immunohistochemical analysis of patient biopsies, by investigating a cellular TB network model and finally by cytokine analysis of BALF from TB patients. We specifically focused on intercellular networks between lymphocytes and airway epithelial cells in TB, and have shown for the first time that lymphocyte‐derived IL‐17 drives both epithelial‐ and fibroblast‐derived MMP‐3 in a TB network, whereas direct infection does not. IL‐17 was expressed in the lymphocytes associated with TB granulomas and MMP‐3 was expressed in the epithelial cells around these TB granulomas. MMP‐3 expression was regulated by the p38 MAP kinase pathway and the proximal (p110α subunit) and distal nodes (AKT) of the PI3K pathway. The effects are MMP‐specific as MMP‐9 secretion was down‐regulated. On examination of BALF from patients with TB, IL‐17 and IL‐22 were both detectable. Several biologics such as monoclonal antibodies that neutralize IL‐17 signalling are now in clinical development 64, 65. In the context of TB, modulating the T_H_‐17 pathway may alter MMP activity, reduce tissue destruction, and improve outcomes. In the present era of increasing anti‐mycobacterial drug resistance, host‐directed therapy is emerging as a key paradigm for future TB treatment 66.

## Author contributions statement

SS designed and performed experiments, analysed data, and prepared the first draft of the manuscript. GMG performed experiments on MRC‐5 fibroblasts. UKS designed and conducted the clinical study. FM performed the immunohistochemical analyses. PTE was involved in experimental design, analysis and interpretation of data, as well as preparation of the final manuscript. JSF was the principal investigator who conceived the project and was responsible for overall direction of the study, interpretation of data, and writing of the final manuscript.

**SUPPLEMENTARY MATERIAL ONLINE**

**Supplementary materials and methods**

**Supplementary figure legends**

**Figure S1.** Transfection efficiency was confirmed with siGLO

**Figure S2.** Positive and negative controls for IL‐17 immunohistochemistry

**Figure S3.** IL‐17 drives concentration‐dependent MMP‐3 secretion in both SAEC and NHBE cells but does not alter TIMP‐1/‐2 secretion

**Figure S4.** Epithelial MMP‐3 and MMP‐9 secretion was not altered by IL‐22 or IL‐23

**Figure S5.** TNF‐α did not increase MMP‐3 secretion from NHBE cells

**Figure S6.** IL‐23 was not detectable in TB or control BALF samples

**Figure S7.** siRNA‐mediated knockdown of p38 and PI3K p110α was confirmed by phospho‐western analysis and by suppression of mRNA expression

**Table S1.** Cytokine and chemokine concentrations in the culture medium of normal human bronchial epithelial cells stimulated with CoMTb or CoMTb + IL‐17

**Table S2.** Cytokine and chemokine concentrations in the culture medium of monocytes stimulated with CoMCont or CoMTb

## Supporting information


**Supplementary materials and methods**

**Supplementary figure legends**

**Figure S1.** **Transfection efficiency was confirmed with siGLO**
72.45% transfection of NHBEs was achieved with siGLO, the transfection control. The dot plot figure demonstrates the upper right and left quadrants only.

**Figure S2.** **Positive and negative controls for IL‐17 immunohistochemistry**
**(A)** As a positive control, colonic T lymphocytes showed strong staining for IL‐17 (scale bar = 200 μm). **(B)** No staining was seen when a secondary antibody only was used as a negative control (scale bar = 100 μm).

**Figure S3.** **IL‐17 drives concentration‐dependent MMP‐3 secretion in both SAEC and NHBE cells but does not alter TIMP‐1/‐2 secretion**
**(A)** SAECs were stimulated with increasing concentrations of IL‐17. MMP‐3 secretion peaked at 30 ng/ml IL‐17, after which it remained unchanged. There was a concentration‐dependent increment in MMP‐3 concentration from a baseline of 145.6 ± 9.9 pg/ml to a maximal concentration of 1575.4 ± 91.44 pg/ml when the cells were stimulated with 30 ng/ml IL‐17. **(B)** IL‐17 did not significantly alter the baseline or CoMTb‐dependent TIMP‐1 suppression from SAECs. **(C)** TIMP‐2 secretion was also unaffected by CoMTb or IL‐17. **(D)** NHBE cells were stimulated with increasing concentrations of IL‐17. MMP‐3 secretion peaked at 10 ng/ml IL‐17. There was a concentration‐dependent increment in MMP‐3 concentration from a baseline of 155.6 ± 10.4 pg/ml to a maximal concentration of 1462.4 ± 292 pg/ml when the cells were stimulated with 10 ng/ml IL‐17.

**Figure S4.** **Epithelial MMP‐3 and MMP‐9 secretion was not altered by IL‐22 or IL‐23**
**(A)** MMP‐3 secretion from NHBEs was unaffected by IL‐22 and also by **(B)** IL‐23. **(C)** MMP‐9 secretion was also unaltered by IL‐22 and **(D)** by IL‐23. These were investigated over a concentration range of 1–30 ng/ml in a TB network.

**Figure S5.** **TNF‐α did not increase MMP‐3 secretion from NHBE cells**
Stimulation of NHBE cells with TNF‐α (concentration range 1–20 ng/ml) in combination with IL‐17 did not drive MMP‐3. TNF‐α alone at a maximal dose of 20 ng/ml also did not drive MMP‐3.

**Figure S6.** **IL‐23 was not detectable in TB or control BALF samples**
IL‐23 was not detectable in the majority of BALF samples from TB and control subjects (n = 17 for TB patients, n = 18 for well‐matched controls).

**Figure S7.** **siRNA‐mediated knockdown of p38 and PI3K p110α was confirmed by phospho‐western analysis and by suppression of mRNA expression**
**(A)** On phospho‐western analysis of p38 in NHBEs, CoMTb and IL‐17‐mediated activation was abrogated by p38‐specific siRNA. A concentration‐dependent response was observed and no activity was seen at 30 nm of the siRNA. **(B)** Total p38 mRNA levels were suppressed to below baseline when the NHBEs were incubated with the p38‐specific siRNA in a concentration‐dependent manner. **(C)** On phospho‐western analysis of PI3K p110α in NHBEs, CoMTb and IL‐17‐mediated activation was abrogated with the specific siRNA. A concentration‐dependent response was again observed and was complete with 30 nm. **(D)** Total p110α mRNA levels were suppressed to below baseline when the NHBEs were incubated with the p110α‐specific siRNA. Non‐targeting siRNA had no effect.

**Table S1.** Cytokine and chemokine concentrations in the culture medium of normal human bronchial epithelial cells stimulated with CoMTb or CoMTb + IL‐17

**Table S2.** Cytokine and chemokine concentrations in the culture medium of monocytes stimulated with CoMCont or CoMTb
