# Supplementary material for: Interleukin‐17 regulates matrix metalloproteinase activity in human pulmonary tuberculosis
Source: J Pathol. 2018 Jan 18;244(3):311–22. doi: 10.1002/path.5013 (PMC5838784; doi:10.1002/path.5013)
Supplement: Supplementary file 1 — Supplementary materials and methods [file PATH-244-311-s001.docx]

**Supplementary materials and methods**

**Reagents**

General laboratory reagents were purchased from Sigma, Poole, UK and Invitrogen, Paisley, UK. Mtb culture reagents were purchased from BD Biosciences, Oxford, UK. Inhibitory chemicals SB203580 (p38 inhibitor), LY294002 (PI3K inhibitor), AKT inhibitor VIII (AKT inhibitor), and phospho- and total-p38 antibodies were purchased from Merck Chemicals Ltd, Nottingham, UK. Recombinant human IL-17, IL-22, and TNF-α were purchased from Peprotech, London, UK. Ficoll Paque and the ECL system were purchased from Amersham Biosciences, Little Chalfont, UK. All siRNA oligonucleotides and reagents were purchased from Thermo Scientific Dharmacon, Cramlington, UK.

**Immunohistochemical analysis of patient biopsies**

Ethical consent for the use of archived lung biopsies was obtained from the Hammersmith Hospitals Research Ethics Committee. Immunohistochemistry was performed on paraffin-embedded lung biopsies from five patients with culture-proven Mtb infection and five uninfected controls. Sections of 4 μm thickness were dewaxed with three changes of xylene followed by three changes of alcohol, before being rehydrated. Endogenous peroxidase activity was blocked with 0.6% hydrogen peroxide for 15 min. Antigen retrieval was performed by microwaving the sections for 20 min in citrate buffer (0.01 m citrate, pH 6.0). Non-specific binding was blocked with 5% normal goat serum for 10 min. The primary antibodies were applied in 0.01 m PBS/azide/BSA for 1 h at room temperature. After three rinses in PBS, the antibody was detected with the Menarini non-biotinylated kit according to the manufacturer’s instructions. Peroxidase activity was developed with diaminobenzidine (DAB, Menarini). Slides were counterstained with Coles haematoxylin, dehydrated, and mounted. Antibodies for MMP-3 and IL-17 were purchased from Abcam, Cambridge, UK (catalogue numbers ab137659 and ab9565, respectively). The antibodies were both polyclonal rabbit and had a human-specific reactivity.

**Clinical study**

BALF samples were collected from patients being routinely investigated for respiratory symptoms at Nalanda University Hospitals, Patna, India. The study was approved by the ethics review board at Nalanda Medical College and University Hospitals (reference SS/0810/TB). To limit user and procedure variability, bronchoscopy was performed by one of two bronchoscopists using flexible bronchoscopes. Samples that were Mtb culture-positive were stored at −20°C. Exclusion criteria from the study were a previous history of TB [to decrease the likelihood of multi- and extensively drug-resistant (MDR/XDR) cases], age less than 18 years, severe chronic lung disease, malignancy, positive HIV status, exposure to corticosteroids or immunosuppressive drugs, or inability to consent. Samples were centrifuged to remove cellular debris and then sterile filtered through a 0.2 μm Durapore membrane (Millipore, Nottingham, UK) to remove Mtb from the samples. This does not interfere with the detection of cytokines. IL-17 concentration was measured with a Bioplex Luminex 200 (Biorad Laboratories Ltd, Hemel Hempstead, UK) and IL-22 with an ELISA kit (R&D Systems Europe, Abingdon, UK).

**Mtb culture and generation of TB medium**

*M. tuberculosis* H37Rv was cultured in Middlebrook 7H9 medium supplemented with 10% albumin, dextrose–catalase enrichment medium, 0.2% glycerol, and 0.02% Tween-80 with agitation. Culture growth was monitored with a Biowave cell density meter (WPA, Cambridge, UK) and the Mtb was sub-cultured when the optical density exceeded 1.00. For infection experiments, culture at mid log growth at an optical density of 0.60 was used, which corresponded to 1 × 10^8^–2 × 10^8^ colony-forming units (CFU) per ml, within a two-fold error. Optical density was correlated with CFU by performing colony counts in triplicate on Middlebrook 7H11 agar supplemented with OADC enrichment medium and 0.5% glycerol. The amebocyte lysate assay (Associates of Cape Cod, East Falmouth, MA, USA) was used to assay the endotoxin level of the Mtb culture and this was found to be less than 0.3 ng/ml lipopolysaccharide.

**Monocyte purification**

Monocytes used in the experiments were from two donor buffy coats, from the National Blood Transfusion Service, Colindale, London, UK. The leukocytes were mixed 50:50 with Hanks’ balanced salt solution (HBSS), layered onto Ficoll Paque (Amersham Biosciences, Little Chalfont, UK), and centrifuged at 480 rcf (relative centrifugal force) for 30 min. The mononuclear cell layer was removed and then washed a total of five times in HBSS, spinning down the cell pellet at 308 rcf after each wash. Total monocytes were calculated by counting the number of adherent cells in a Neubauer counting chamber after incubation for 5 min at 37^o^C. Monocytes were plated in at 250 000 monocytes/cm^2^ diluted in RPMI 1640. After 1 h, non-adherent cells were removed by washing three times with HBSS; then the media were replaced with RPMI 1640 supplemented with 2 mm glutamine and 10 μg/ml ampicillin. Monocyte purity was assessed using two-colour fluorescence-activated cell scanning (FACS) for anti-CD3 (fluorescein isothiocyanate, FITC; BD Pharmingen, Oxford, UK) and anti-CD14 (phycoerythrin, PE; BD Pharmingen) and analysed on a BD FACSCaliber flow cytometer. Monocyte purity was over 95%, with less than 5% cells CD3-positive.

**Monocyte and epithelial cell infection protocol**

Monocytes were cultured in RPMI with 2 mm glutamine and 10 μg/ml ampicillin (which has been shown not to inhibit the growth of mycobacteria at this concentration). Monocytes were infected immediately after the completion of adhesion purification, using Mtb at an OD of 0.60 to calculate the correct multiplicity of infection (MOI). Mtb was sonicated for 30 s prior to infection to minimize clumping. Trypan blue exclusion was used to compare cell viability in uninfected and infected monocytes at 24 h and no differences were observed. For epithelial cell infections, media were changed at the start of the experiment and Mtb was added at the appropriate MOI. Cells were then washed after 2 h to remove non-adherent bacilli and fresh culture was medium added. Ziehl-Nielsen staining demonstrated that 30% of cells were infected at 6 h at an MOI of 10, which is similar to reports of A549 infection rates. Mtb was removed by filtration through a 0.2 μm Durapore membrane.

**Generation of CoMTb**

Cell culture medium was harvested at 24 h. Medium was spun at 13 000 rcf to remove cellular debris and then sterile filtered through a 0.2 µm Anopore membrane. Colony counting on Middlebrook 7H11 agar showed no viable bacteria in CoMTb. Medium from infected monocytes was termed conditioned medium from monocytes infected with Mtb (CoMTb). With each batch of CoMTb that was generated, supernatants were also collected from uninfected monocytes (CoMCont). A comparative analysis of the cytokines and chemokines in CoMCont and CoMTb has been performed previously using the Luminex Bead Multi-Analyte Profiling facility. Monocytes infected with Mtb consistently secrete more cytokines and chemokines than uninfected monocytes (supplementary material, Table S2). Donor-related variability in the cytokine and chemokine profiles of CoMTb was within statistically acceptable limits. Both CoMCont and CoMTb were used at a dilution of 1 in 5 (with the cell culture medium) in 12- or 24-well plates.

**Cell culture**

Primary small airway epithelial cells (SAECs) and primary normal human bronchial epithelial (NHBE) cells were purchased from Lonza Biosciences, Basel, Switzerland. Cells had been acquired bronchoscopically from healthy male adult volunteers. Each frozen ampoule contained ~0.5–1 × 10^6^ cells. Cells were initially propagated in a T25 flask until they reached 70–80% confluence. On subsequent subculture, flasks were seeded at 3500 cells/cm^2^ density and after the final subculture, cells were seeded at a density of 1.5 × 10^3^ cells/cm^2^ in a 12- or 24-well plate in fresh medium. Cells were cultured in bronchial epithelial growth media according to the supplier’s instructions. All experiments were performed between passages 4 and 5. For subculture, medium was aspirated; the cell monolayer was washed with hydroxyethyl piperazine ethanesulfonic acid (HEPES) solution; and trypsin was added for 5 min and then neutralized with trypsin neutralizing solution.

The human MRC-5 fibroblast line was grown in Eagle’s medium with 10% fetal calf serum (FCS) and subcultured when cells were 70–80% confluent as per the suppliers’ instructions (Sigma-Aldrich, Gillingham, Dorset, UK). Adherent cells were washed with PBS and then detached from the surface with 0.25% trypsin-ethylenediaminetetraacetic acid (EDTA) solution. Cells were resuspended in fresh media at a seeding density of 2–4 × 10^4^ cells/cm^2^. For experiments, 1–2 × 10^4^ cells/cm^2^ were seeded in a 24-well plate in fresh medium with 1% FCS and stimulated at 70–80% confluence.

**Experimental design**

When epithelial cells were 80% confluent, they were stimulated with a 1 in 5 dilution of conditioned medium from monocytes infected with Mtb (CoMTb), as previous studies had shown maximal MMP response at this dilution. For MRC-5 cells, CoMTb was used at a 1 in 50 dilution. Cells were pretreated for 2 h with chemical inhibitors which were used at the concentrations specified in the graphs. The inhibitors were dissolved in dimethyl sulfoxide (DMSO), which was also added to the control wells (maximal final concentration 0.1%). After the use of CoMTb and inhibitors, the viability of cells was confirmed and no differences were observed in cells treated with inhibitor. Supernatants were harvested at 72 h after stimulation for MMP secretion analysis and mRNA extraction was performed at 24 h after stimulation. For kinetic experiments, supernatants or RNA extraction was performed at 0, 24, 48, 72, and 96 h. Supernatants were spun at 11 700 rcf for 5 min to remove cellular debris and then frozen at −20°C. Baseline levels of MMPs in the conditioned media used to stimulate cells were negligible compared with the concentrations secreted by the stimulated cells as the CoMCont and CoMTb were taken from monocytes at 24 h, whereas monocyte MMP secretion increases from 48 h and peaks at 72 h.

**Measurement of MMP and TIMP concentrations**

MMP-1, -3, -9 and TIMP-1, -2 concentrations in cell culture medium were measured by ELISA according to the manufacturer’s instructions (R&D Systems Europe, Abingdon, UK). The lower level of detection was 30 pg/ml. MMP-1, -3 and -9 concentrations were also analysed using a Fluorokine multianalyte profiling kit according to the manufacturer’s protocol (R&D Systems Europe) on the Luminex platform (Bio-Rad Laboratories Ltd, Hemel Hempstead, UK). The minimum level of detection for MMPs was 10 pg/ml.

**Gelatin zymography**

MMP-9 gelatinolytic activity was detected by zymography using standard methodology. In brief, standards and cell culture supernatants were loaded with 5× loading buffer [0.25 m Tris (pH 6.8), 50% glycerol, 5% sodium dodecyl sulphate (SDS), and bromophenol blue] and run on 11% acrylamide gels impregnated with 0.1% gelatin as substrate. After 3.5 h at 180 V (buffer 25 mm Tris, 190 mm glycine, and 0.1% SDS), the gel was renatured in 2.5% Triton X for 1 h with agitation. After two washes in collagenase buffer (55 mm Tris base, 200 mm sodium chloride, 5 mm calcium chloride, and 0.02% Brij, pH 7.6), gels were incubated overnight in fresh collagenase buffer at 37°C. Gelatinolytic activity was detected using 0.02% Coomassie blue in 1:3:6 acetic acid–methanol–water. All experimental samples were run in parallel with 2 ng of recombinant MMP-9 standard (Merck Chemicals Ltd, Nottingham, UK). Digital image acquisition (UVP, Ultraviolet Products) of the bands was followed by densitometric analysis using the image processing software Scion Image (Scion Corporation Scientific Computing, Frederick, MD, USA).

**Promoter-reporter assay**

Promoter-reporter studies were performed using FuGENE^®^ HD Transfection Reagent from Roche Applied Sciences (Sigma-Aldrich, Gillingham, Dorset, UK) and Promega’s Dual-Luciferase^™^ Reporter (DLR^™^) Assay System (Promega UK, Southampton, UK). The MMP-3 promoter (1206 base pairs) linked to firefly luciferase in pGL4 basic vector (Promega) and the reference gene thymidine kinase promoter linked to *Renilla* luciferase in the pRL control vector (pRL-TK; Promega) were generated. MRC-5 fibroblasts were grown overnight in a 12-well plate (3.8 cm^2^/well) at a recommended seeding density of 100 000 cells per well so that the monolayer was 80–90% confluent at the time of transfection. A 6:2 ratio of transfection reagent (37.6 µl/well) to purified plasmid DNA (1.6 µg/well) and pRL-TK (0.16 µg/well) was used according to the manufacturer’s instructions. Plasmid DNA purity and concentration had been estimated using a 260 nm/280 nm absorbance ratio, the optimal ratio being 1.8. Fibroblasts were stimulated with 10% CoMTb and harvested at given time intervals (0, 6, 24, and 48 h). Cells were washed twice with sterile PBS prior to using 100 µl of passive lysis buffer from the DLR^™^ Assay kit (Promega). Luminescence was detected using the Promega Dual Luciferase Assay system. *Renilla* luciferase activity was used to normalize firefly activity in order to control for transfection efficiency. Results were expressed as relative luminescence (RLU) for firefly/*Renilla* luciferase (ratio of luminescence for the MMP-3 promoter to that of the thymidine kinase promoter).

**Phospho-western analysis**

NHBE cells were stimulated in six-well plates and at specific time points, cells were washed with sterile phosphate-buffered saline (PBS), lysed with 100 µl of SDS sample buffer [62.5 mm Tris (pH 6.8), 2% SDS, 10% glycerol, 50 mm dithiothreitol (DTT), and 0.01% bromophenol blue], transferred to cold microtubes, and frozen at −80ºC. Forty-microlitre samples were mixed with 40 µl of loading buffer [10% glycerol, 5% 2-mercaptoethanol, 2% SDS, 0.06 m Tris (pH 6.8), bromophenol blue] and heat-inactivated at 100ºC for ~2 min prior to separation on 10% acrylamide gels. Molecular weight markers (Amersham Biosciences, Little Chalfont, UK) were run concurrently. Subsequently, proteins were electro-transferred to a nitrocellulose membrane (Amersham Biosciences), which was blocked for 1 h with agitation in 5% milk protein (Marvel, Nestlé) mixed with 0.1% Tween-20. The membrane was probed with a primary antibody by incubating at 4°C and the next day, the membrane was washed three times and incubated for 1 h with HRP-linked goat anti-rabbit IgG secondary antibody. Luminescence was then detected with the ECL system according to the manufacturer’s protocol (Sigma-Aldrich).

**Small interfering (siRNA) transfection**

All siRNA oligonucleotides and reagents were purchased from Thermo Scientific Dharmacon. The siRNAs were purchased as double-stranded, chemically synthesized oligonucleotides in a smart pool, targeting the transcription products from four alleles of the gene of interest. For reconstitution, the siRNA was dissolved in a special buffer, mixed on a shaker for 30 min, aliquoted, and then stored at −80°C. Prior to planning the individual experiments, the conditions were optimized using a transfection control. The transfection control, siGLO (Green), confirms localization to the nucleus and optimal delivery of the siRNA in the cell type being investigated. It is an oligonucleotide labelled with a fluorophore on the sense strand. Figure S1 (supplementary material) illustrates the 72.45% transfection achieved in NHBE cells with siGLO at 30 nm in a 1:1 ratio with Lipofectamine. A negative control of non-targeting sequences was incorporated in all experiments to distinguish sequence-specific silencing from non-specific effects of siRNA. In brief, 12-well plates were seeded with NHBE cells at 150 000 cells per well the day before experiments. Lipofectamine was used at 25 μg/ml per well, and siRNA at 10–30 nm per well. Lipofectamine and siRNA were allowed to complex in a 1:1 ratio (by volume) at room temperature for 20 min. The mixture was then added to cells with the basal medium for 4 h. Cells were then washed, rested for an additional 4 h, and then stimulated. Samples were collected at the end of the experiment.

**RNA extraction, cDNA synthesis, and reverse transcription–quantitative polymerase chain reaction (RT-qPCR)**

RNA extraction was performed using the Qiagen RNeasy Minikit according to the manufacturer’s instructions (Qiagen, Manchester, UK). RNA was eluted with RNase-free water and stored at −80°C. cDNA synthesis was performed using the Quantitect reverse transcription kit. RNA was quantified on a Nanodrop spectrophotometer. A volume of RNA equivalent to 1 μg was diluted to 12 µl with RNase-free water. Two microlitres of genomic (g) DNA wipeout buffer was added to each sample. Samples were then heated at 42°C for 2 min. Six microlitres of the reverse transcription master mix was added to each sample, before heating again at 42°C for 15 min, followed by 95°C for 3 min. The resulting cDNA was stored at −20°C. Real-time quantitative PCR was performed using Brilliant II QPCR master mix (Stratagene, Cambridge, UK) on the Stratagene Mx3000P platform. MMP primers and probes were as described previously. The C_T_ at which amplification entered the exponential phase was determined. A lower C_T_ indicates a higher quantity of starting RNA. To determine the relative RNA levels within samples, standard curves were prepared by making five-fold serial dilutions of each sample. Standard curves for C_T_ versus input RNA were prepared and relative quantities of starting RNA in each sample were determined. Experimental MMP data were normalized to three reference RNAs, namely, *GAPDH*, *18S* ribosomal RNA, and cyclophilin A (*PPIA*), whose C_T_ values remained stable under different experimental conditions. Analysis of *MMP* mRNA expression was first undertaken by the standard curve method and results were corroborated by using the C_T_ values to assess the levels of gene expression.

**Statistical analysis**

Data are presented as mean ± SD and represent experiments performed in triplicate on at least two occasions, unless stated otherwise. Paired groups were compared using Student’s *t*-test. Multiple intervention experiments were compared by two-way ANOVA, with Tukey’s correction for multiple pairwise comparisons. BALF MMP concentrations were compared using the Mann–Whitney *U*-test. A *p* value of less than 0.05 was taken as statistically significant. In figures, *p* < 0.05 is illustrated with *, *p* < 0.01 with **, *p* < 0.001 with ***, and *p* < 0.0001 with ****. All secretion and mRNA expression data shown here are representative of three separate experiments.
