# Supplementary material for: Interleukin‐17 regulates matrix metalloproteinase activity in human pulmonary tuberculosis
Source: J Pathol. 2018 Jan 18;244(3):311–22. doi: 10.1002/path.5013 (PMC5838784; doi:10.1002/path.5013)
Supplement: Supplementary file 2 — Supplementary figure legends [file PATH-244-311-s002.doc]

**Supplementary figure legends**

**Figure S1. Transfection efficiency was confirmed with siGLO**72.45% transfection of NHBEs was achieved with siGLO, the transfection control. The dot plot figure demonstrates the upper right and left quadrants only.

**Figure S2. Positive and negative controls for IL-17 immunohistochemistry
(A)** As a positive control, colonic T lymphocytes showed strong staining for IL-17 (scale bar = 200 μm). **(B)** No staining was seen when a secondary antibody only was used as a negative control (scale bar = 100 μm).

**Figure S3. IL-17 drives concentration-dependent MMP-3 secretion in both SAEC and NHBE cells but does not alter TIMP-1/-2 secretion
(A)** SAECs were stimulated with increasing concentrations of IL-17. MMP-3 secretion peaked at 30 ng/ml IL-17, after which it remained unchanged. There was a concentration-dependent increment in MMP-3 concentration from a baseline of 145.6 ± 9.9 pg/ml to a maximal concentration of 1575.4 ± 91.44 pg/ml when the cells were stimulated with 30 ng/ml IL-17. **(B)** IL-17 did not significantly alter the baseline or CoMTb-dependent TIMP-1 suppression from SAECs. **(C)** TIMP-2 secretion was also unaffected by CoMTb or IL-17. **(D)** NHBE cells were stimulated with increasing concentrations of IL-17. MMP-3 secretion peaked at 10 ng/ml IL-17. There was a concentration-dependent increment in MMP-3 concentration from a baseline of 155.6 ± 10.4 pg/ml to a maximal concentration of 1462.4 ± 292 pg/ml when the cells were stimulated with 10 ng/ml IL-17.

**Figure S4. Epithelial MMP-3 and MMP-9 secretion was not altered by IL-22 or IL-23
(A)** MMP-3 secretion from NHBEs was unaffected by IL-22 and also by **(B)** IL-23. **(C)** MMP-9 secretion was also unaltered by IL-22 and **(D)** by IL-23. These were investigated over a concentration range of 1–30 ng/ml in a TB network.

**Figure S5. TNF-α did not increase MMP-3 secretion from NHBE cells**Stimulation of NHBE cells with TNF-α (concentration range 1–20 ng/ml) in combination with IL-17 did not drive MMP-3. TNF-α alone at a maximal dose of 20 ng/ml also did not drive MMP-3.

**Figure S6. IL-23 was not detectable in TB or control BALF samples**IL-23 was not detectable in the majority of BALF samples from TB and control subjects (*n* = 17 for TB patients, *n* = 18 for well-matched controls).

**Figure S7. siRNA-mediated knockdown of p38 and PI3K p110α was confirmed by phospho-western analysis and by suppression of mRNA expression** **(A)** On phospho-western analysis of p38 in NHBEs, CoMTb and IL-17-mediated activation was abrogated by p38-specific siRNA. A concentration-dependent response was observed and no activity was seen at 30 nm of the siRNA. **(B)** Total *p38* mRNA levels were suppressed to below baseline when the NHBEs were incubated with the p38-specific siRNA in a concentration-dependent manner. **(C)** On phospho-western analysis of PI3K p110α in NHBEs, CoMTb and IL-17-mediated activation was abrogated with the specific siRNA. A concentration-dependent response was again observed and was complete with 30 nm. **(D)** Total p110α mRNA levels were suppressed to below baseline when the NHBEs were incubated with the p110α-specific siRNA. Non-targeting siRNA had no effect.
