# Supplementary figures and images for: Interleukin‐17 regulates matrix metalloproteinase activity in human pulmonary tuberculosis
Source: J Pathol. 2018 Jan 18;244(3):311–22. doi: 10.1002/path.5013 (PMC5838784; doi:10.1002/path.5013)

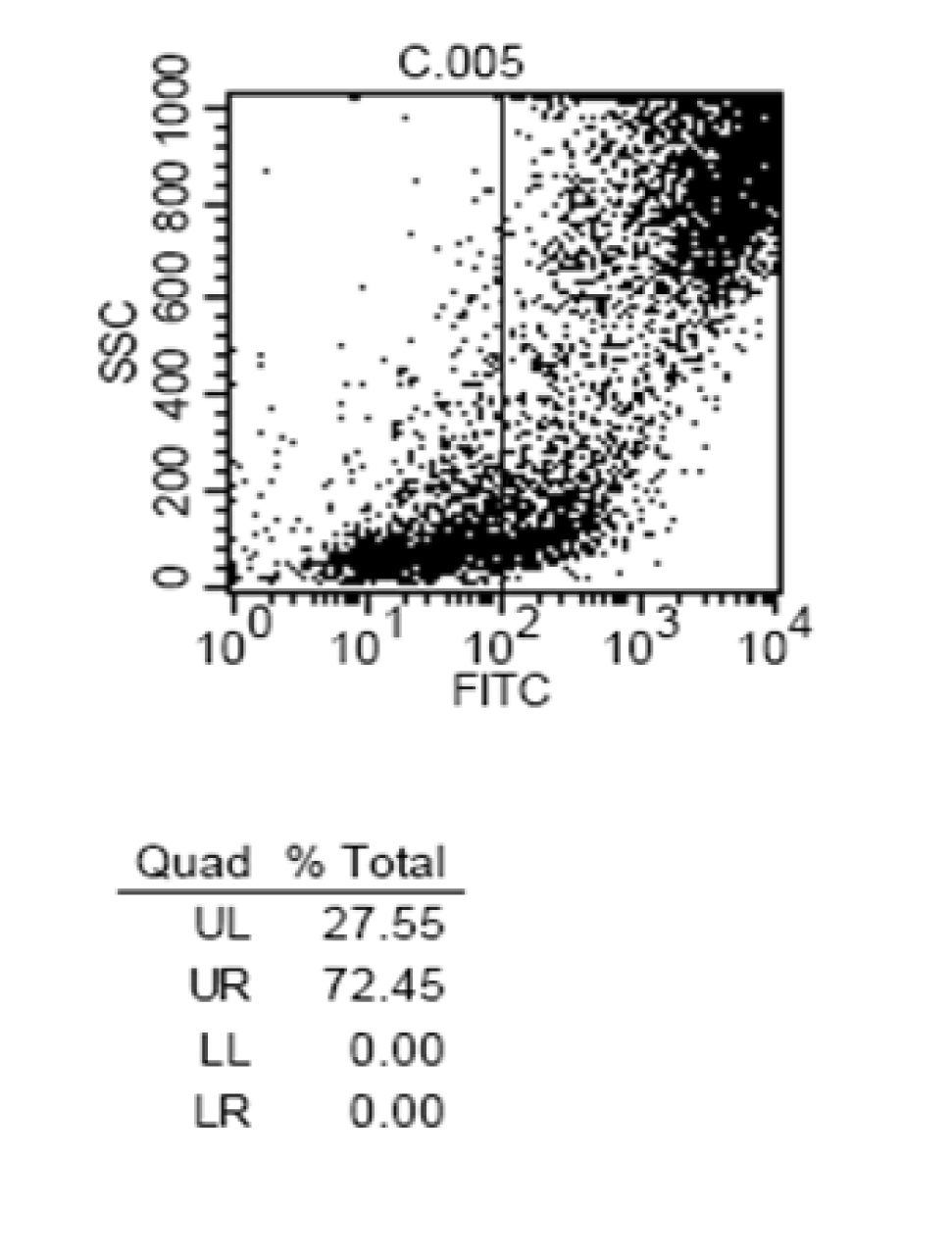

Supplement: Supplementary file 3 — Figure S1. Transfection efficiency was confirmed with siGLO 72.45% transfection of NHBEs was achieved with siGLO, the transfection control. The dot plot figure demonstrates the upper right and left quadrants only. [file PATH-244-311-s003.tif]

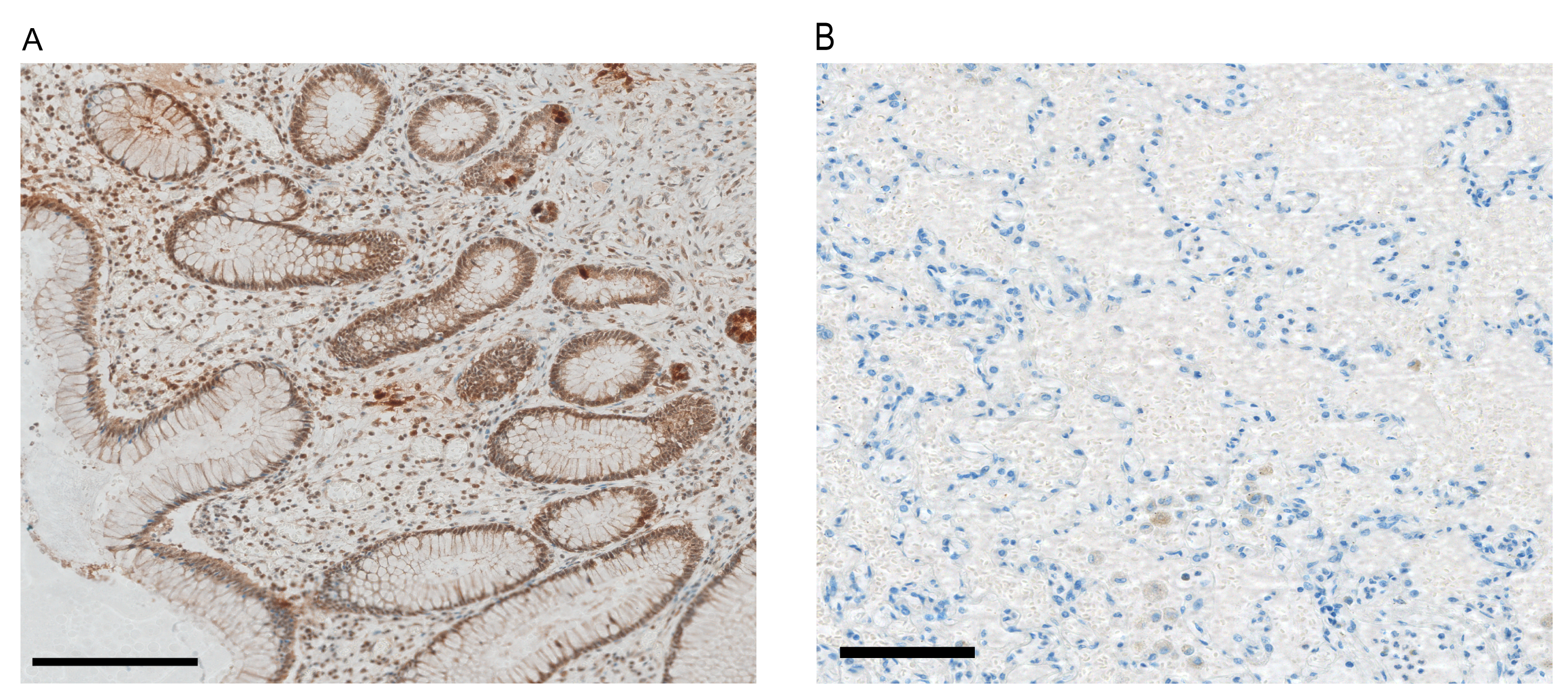

Supplement: Supplementary file 4 — Figure S2. Positive and negative controls for IL‐17 immunohistochemistry (A) As a positive control, colonic T lymphocytes showed strong staining for IL‐17 (scale bar = 200 μm). (B) No staining was seen when a secondary antibody only was used as a negative control (scale bar = 100 μm). [file PATH-244-311-s004.tif]

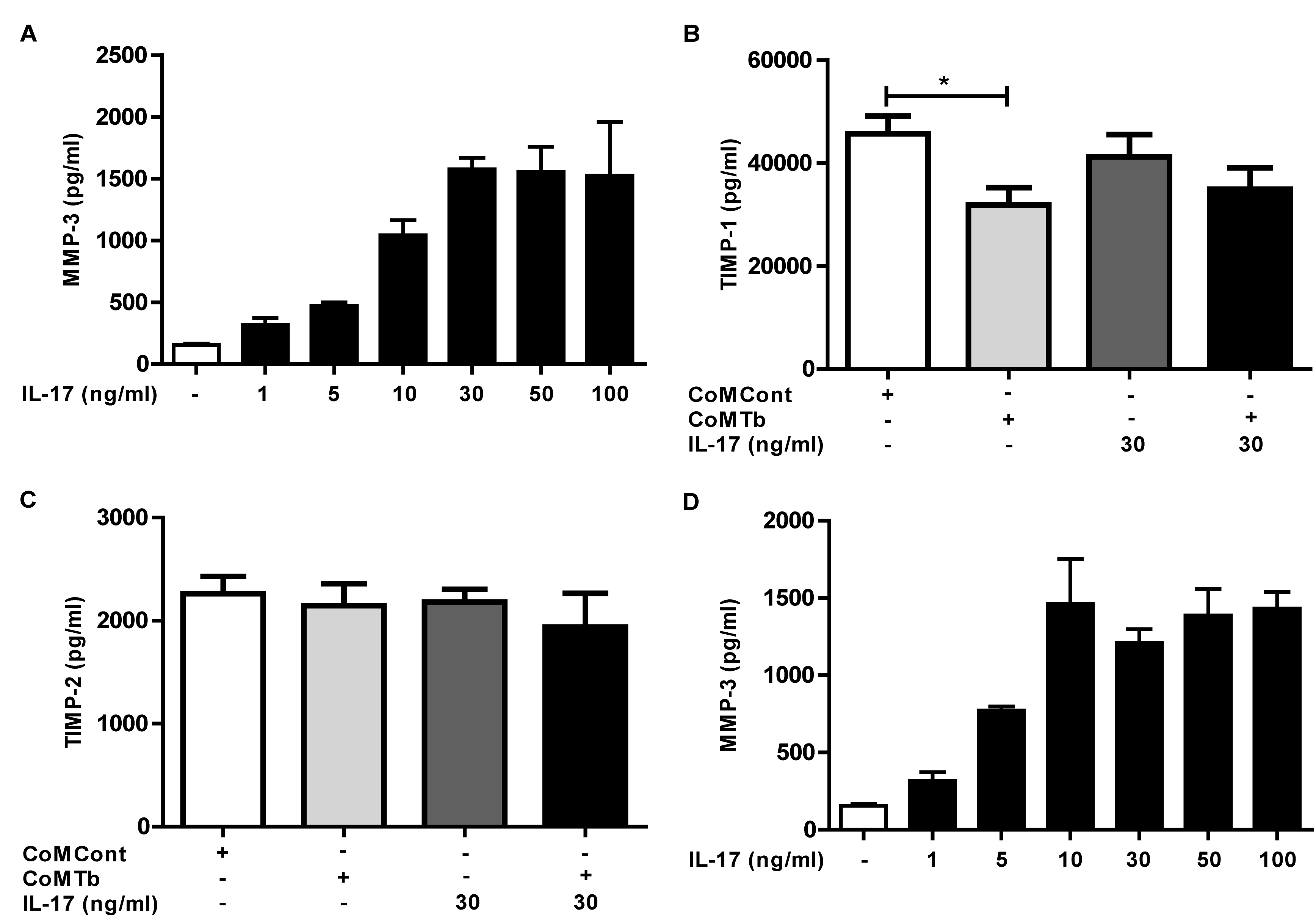

Supplement: Supplementary file 5 — Figure S3. IL‐17 drives concentration‐dependent MMP‐3 secretion in both SAEC and NHBE cells but does not alter TIMP‐1/‐2 secretion (A) SAECs were stimulated with increasing concentrations of IL‐17. MMP‐3 secretion peaked at 30 ng/ml IL‐17, after which it remained unchanged. There was a concentration‐dependent increment in MMP‐3 concentration from a baseline of 145.6 ± 9.9 pg/ml to a maximal concentration of 1575.4 ± 91.44 pg/ml when the cells were stimulated with 30 ng/ml IL‐17. (B) IL‐17 did not significantly alter the baseline or CoMTb‐dependent TIMP‐1 suppression from SAECs. (C) TIMP‐2 secretion was also unaffected by CoMTb or IL‐17. (D) NHBE cells were stimulated with increasing concentrations of IL‐17. MMP‐3 secretion peaked at 10 ng/ml IL‐17. There was a concentration‐dependent increment in MMP‐3 concentration from a baseline of 155.6 ± 10.4 pg/ml to a maximal concentration of 1462.4 ± 292 pg/ml when the cells were stimulated with 10 ng/ml IL‐17. [file PATH-244-311-s005.tif]

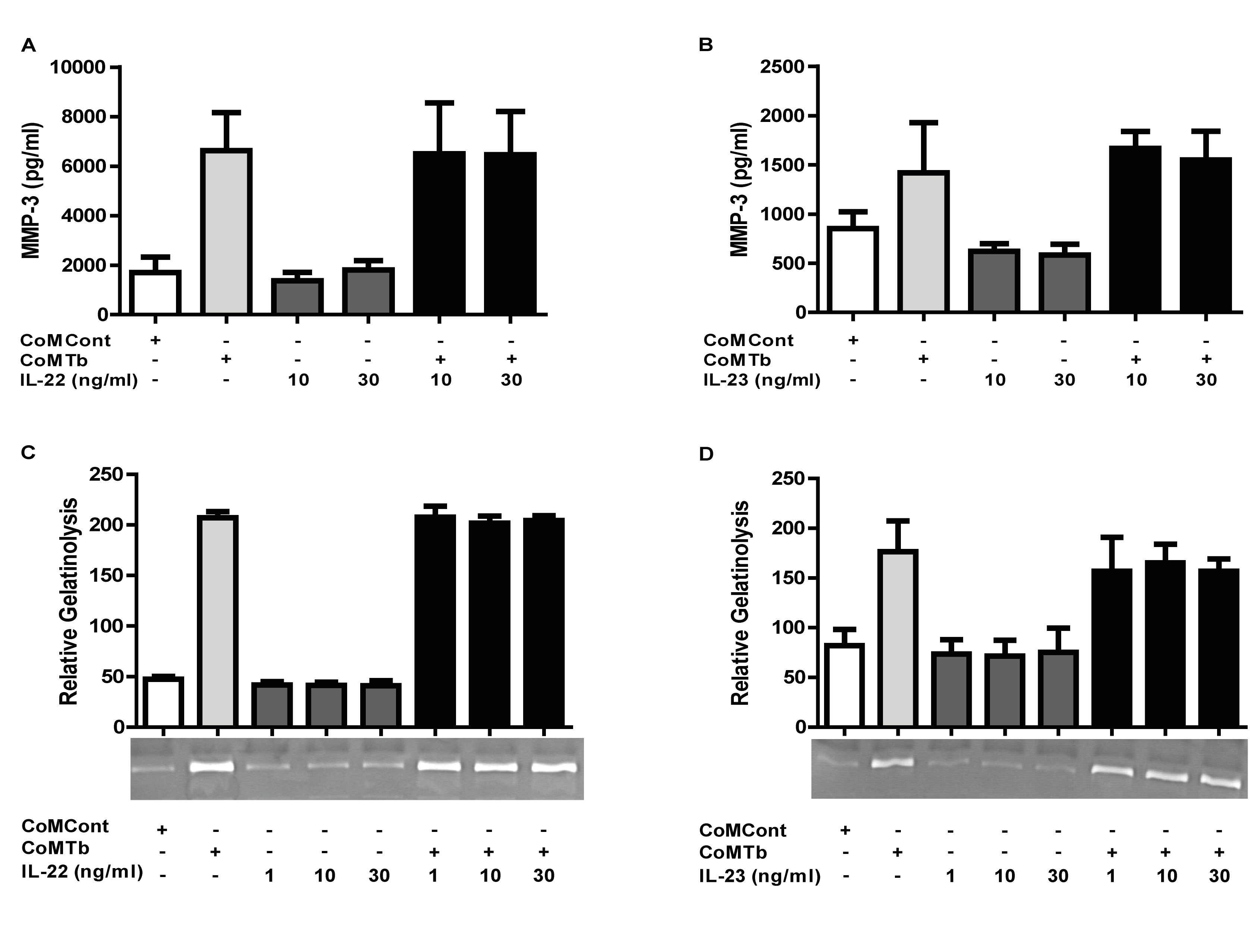

Supplement: Supplementary file 6 — Figure S4. Epithelial MMP‐3 and MMP‐9 secretion was not altered by IL‐22 or IL‐23 (A) MMP‐3 secretion from NHBEs was unaffected by IL‐22 and also by (B) IL‐23. (C) MMP‐9 secretion was also unaltered by IL‐22 and (D) by IL‐23. These were investigated over a concentration range of 1–30 ng/ml in a TB network. [file PATH-244-311-s006.tif]

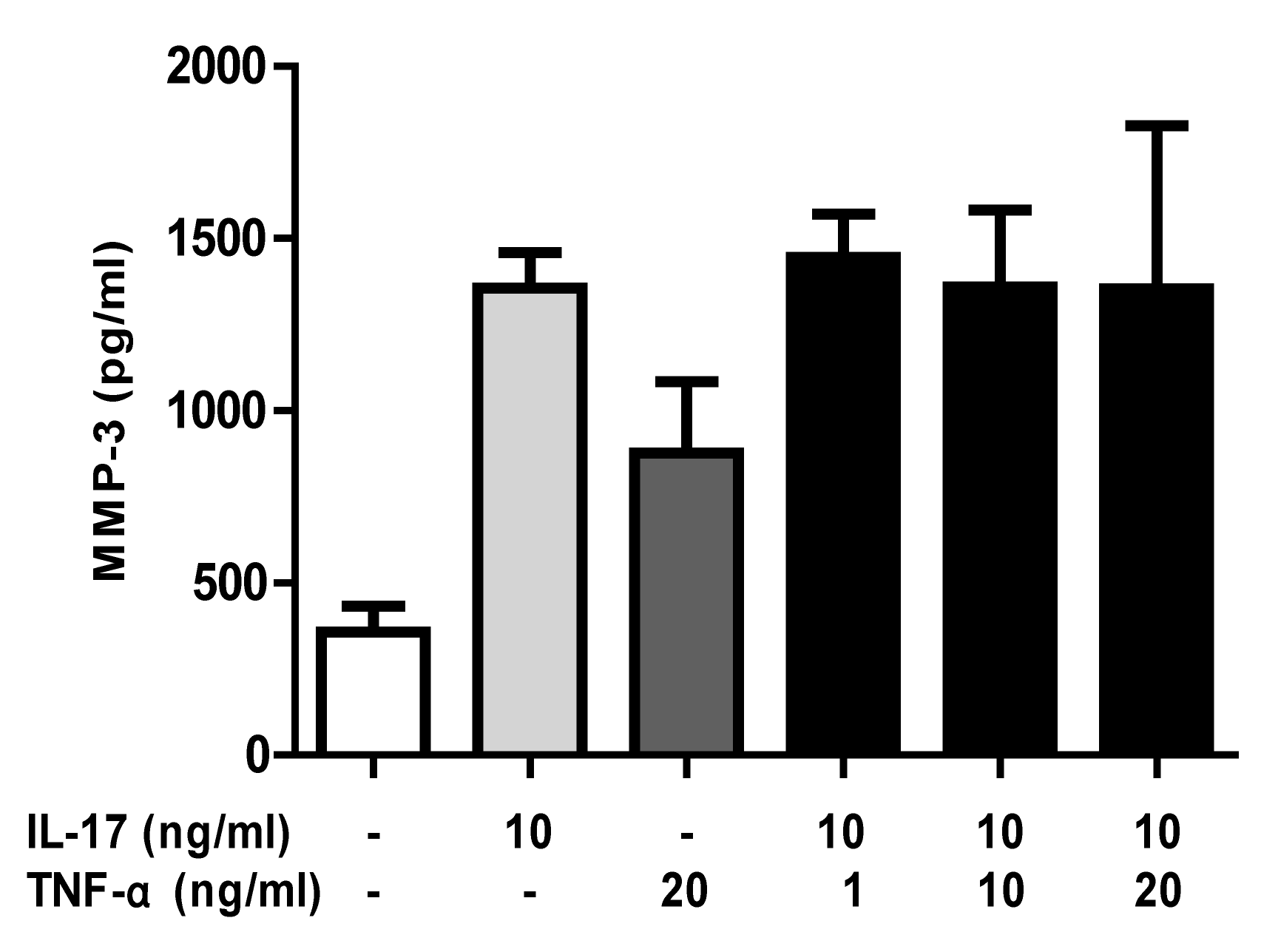

Supplement: Supplementary file 7 — Figure S5. TNF‐α did not increase MMP‐3 secretion from NHBE cells Stimulation of NHBE cells with TNF‐α (concentration range 1–20 ng/ml) in combination with IL‐17 did not drive MMP‐3. TNF‐α alone at a maximal dose of 20 ng/ml also did not drive MMP‐3. [file PATH-244-311-s007.tif]

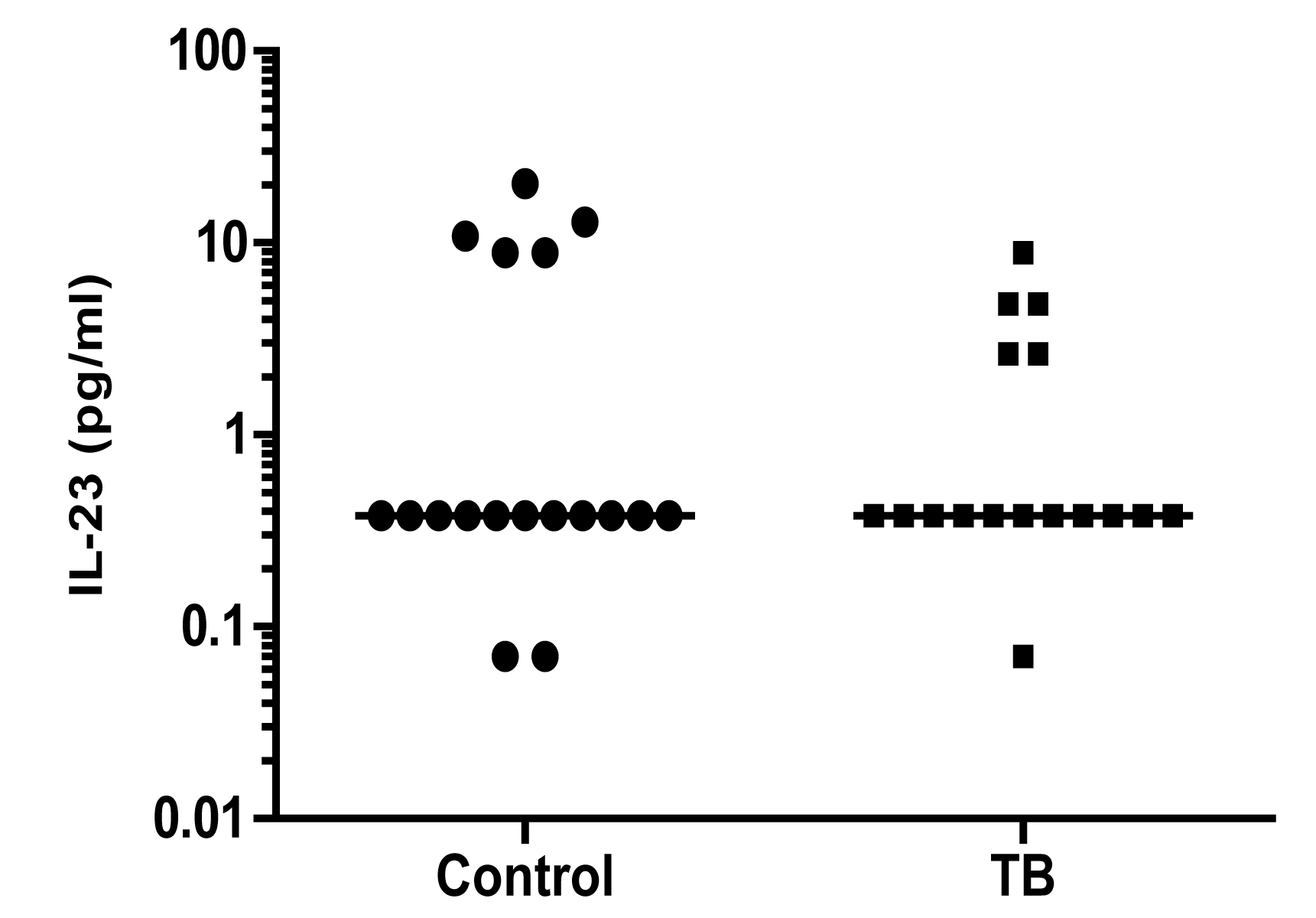

Supplement: Supplementary file 8 — Figure S6. IL‐23 was not detectable in TB or control BALF samples IL‐23 was not detectable in the majority of BALF samples from TB and control subjects (n = 17 for TB patients, n = 18 for well‐matched controls). [file PATH-244-311-s008.tif]

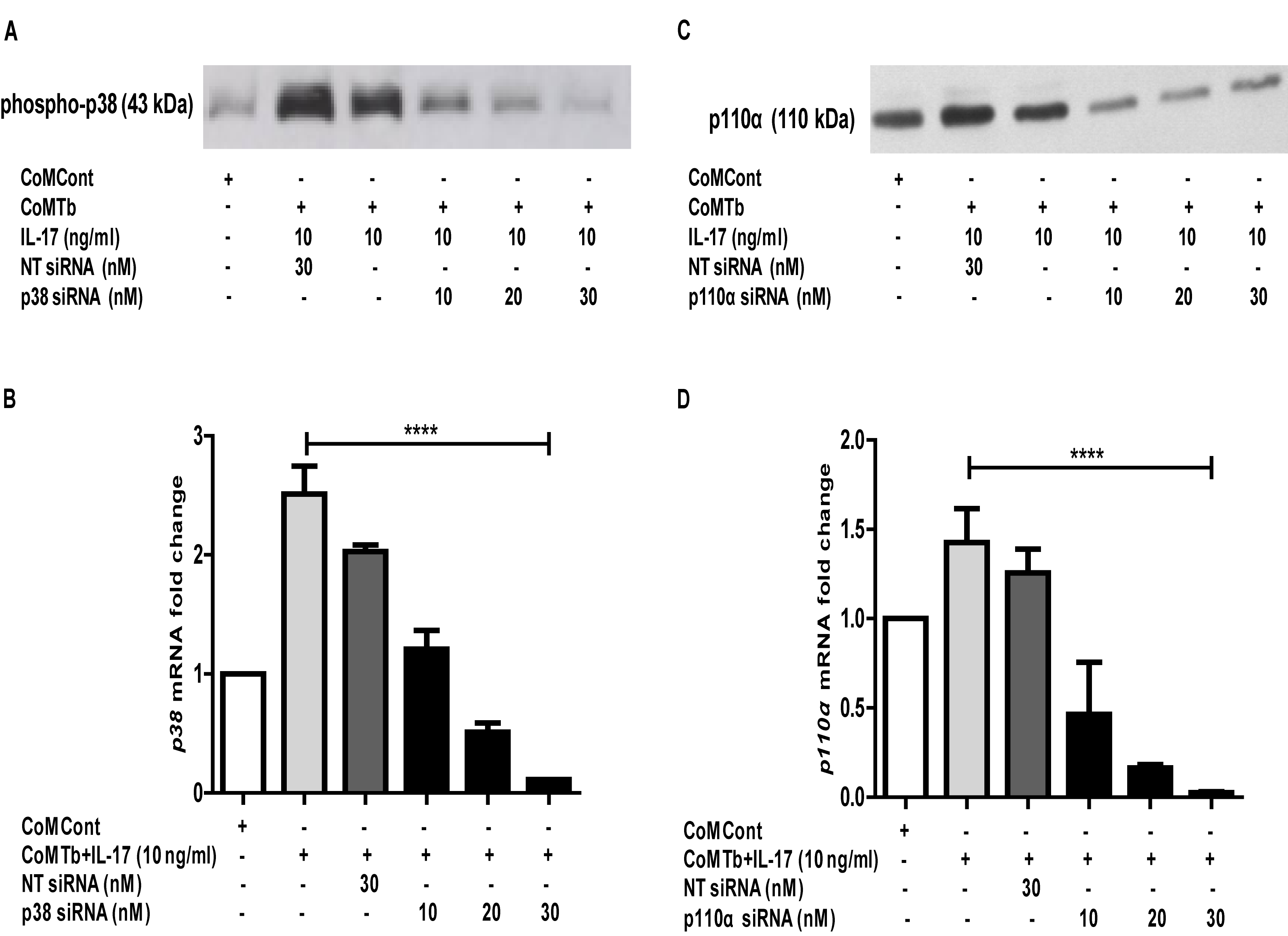

Supplement: Supplementary file 9 — Figure S7. siRNA‐mediated knockdown of p38 and PI3K p110α was confirmed by phospho‐western analysis and by suppression of mRNA expression (A) On phospho‐western analysis of p38 in NHBEs, CoMTb and IL‐17‐mediated activation was abrogated by p38‐specific siRNA. A concentration‐dependent response was observed and no activity was seen at 30 nm of the siRNA. (B) Total p38 mRNA levels were suppressed to below baseline when the NHBEs were incubated with the p38‐specific siRNA in a concentration‐dependent manner. (C) On phospho‐western analysis of PI3K p110α in NHBEs, CoMTb and IL‐17‐mediated activation was abrogated with the specific siRNA. A concentration‐dependent response was again observed and was complete with 30 nm. (D) Total p110α mRNA levels were suppressed to below baseline when the NHBEs were incubated with the p110α‐specific siRNA. Non‐targeting siRNA had no effect. [file PATH-244-311-s009.tif]
