# Supplementary material for: Interleukin‐17 regulates matrix metalloproteinase activity in human pulmonary tuberculosis
Source: J Pathol. 2018 Jan 18;244(3):311–22. doi: 10.1002/path.5013 (PMC5838784; doi:10.1002/path.5013)
Supplement: Supplementary file 10 — Table S1. Cytokine and chemokine concentrations in the culture medium of normal human bronchial epithelial cells stimulated with CoMTb or CoMTb + IL‐17 [file PATH-244-311-s010.docx]

**Table S1.** Cytokine and chemokine concentrations in the culture medium of normal human bronchial epithelial cells stimulated with CoMTb or CoMTb + IL-17

| **Cytokine/chemokine** | **Mean concentration (pg/ml) in NHBEs stimulated with CoMTb** | **Mean concentration (pg/ml) in NHBEs stimulated with CoMTb + IL-17** | ***p* value** |
| --- | --- | --- | --- |
| TNF-α | 611 | 752 | ns |
| IL-1RA | 1774 | 2251 | ns |
| MIP-1α | 5896 | 5951 | ns |
| CXCL-8 | 178 708 | 409 251 | < 0.001 |

CXCL-8 concentration was up-regulated when NHBEs were stimulated with CoMTb and IL-17, compared with CoMTb alone (*p* < 0.001). TNF-α, IL-1RA, and MIP-1α levels were similar in both groups.
