# Supplementary material for: Interleukin‐17 regulates matrix metalloproteinase activity in human pulmonary tuberculosis
Source: J Pathol. 2018 Jan 18;244(3):311–22. doi: 10.1002/path.5013 (PMC5838784; doi:10.1002/path.5013)
Supplement: Supplementary file 11 — Table S2. Cytokine and chemokine concentrations in the culture medium of monocytes stimulated with CoMCont or CoMTb [file PATH-244-311-s011.docx]

**Table S2.** Cytokine and chemokine concentrations in the culture medium of monocytes stimulated with CoMCont or CoMTb

|  | **CoMCont** | | **CoMTb** | |  |
| --- | --- | --- | --- | --- | --- |
| **Cytokine/chemokine** | **Mean (pg/ml)** | **SEM** | **Mean (pg/ml)** | **SEM** | ***p* value** |
| GM-CSF | 3 | 0 | 559.8 | 374 | < 0.0001 |
| G-CSF | 3 | 0 | 1178 | 446.2 | < 0.0001 |
| IL1-β | 131.8 | 44.5 | 26 274 | 8815 | < 0.0001 |
| IL-6 | 1 | 0 | 41 992 | 14 227 | < 0.0001 |
| TNF-α | 1 | 0 | 785 | 303 | < 0.0001 |
| IFN-γ | Undetectable | N/A | Undetectable | N/A | N/A |
| IL-12 | 2 | 0 | 1591 | 1072 | < 0.0001 |
| IL1-RA | 505.5 | 171.7 | 7970 | 2032 | 0.0020 |
| CXCL-8 | 27.75 | 8.9 | 94 865 | 49 375 | < 0.0001 |
| MIP1-α | 4 | 0 | 198 693 | 94 784 | < 0.0001 |
| MCP-1 | 7.25 | 1.25 | 1426 | 642.4 | < 0.0001 |
| MIG | 47 | 22 | 147.3 | 48.2 | ns |
| IL-17 | Undetectable | N/A | Undetectable | N/A | N/A |

Monocytes infected with Mtb (CoMTb) secreted significantly more GM-CSF, G-CSF, IL1-β, IL-6, TNF-α, IL-12, IL1-RA, and chemokines than uninfected monocytes (CoMCont). IL-17 was undetectable in CoMTb.
